# Insights from Structure-Based Simulations into the Persulfidation of Uridine Diphosphate-Glycosyltransferase71c5 Facilitating the Reversible Inactivation of Abscisic Acid

**DOI:** 10.3390/ijms25179679

**Published:** 2024-09-06

**Authors:** Miaomiao Li, Lihui Xiao, Ke Sun, Taotao Qiu, Sisong Lai, Guojing Chen, Lingxi Geng, Siqi Huang, Yanjie Xie

**Affiliations:** 1 College of Life Sciences, Nanjing Agricultural University, Nanjing 210095, China; 2021216040@stu.njau.edu.cn (M.L.); 2023116078@stu.njau.edu.cn (L.X.); 9201010221@stu.njau.edu.cn (L.G.); 2 Institute of Bast Fiber Crops, Chinese Academy of Agricultural Sciences (IBFC, CAAS), Changsha 410221, China

**Keywords:** UGT, glycosylation, ABA, H_2_S, persulfidation, molecular dynamics

## Abstract

The action of abscisic acid (ABA) is closely related to its level in plant tissues. Uridine diphosphate-glycosyltransferase71c5 (UGT71C5) was characterized as a major UGT enzyme to catalyze the formation of the ABA-glucose ester (ABA-GE), a reversible inactive form of free ABA in *Arabidopsis thaliana* (thale cress). UGTs function in a mode where the catalytic base deprotonates an acceptor to allow a nucleophilic attack at the anomeric center of the donor, achieving the transfer of a glucose moiety. The proteomic data revealed that UGT71C5 can be persulfidated. Herein, an experimental method was employed to detect the persulfidation site of UGT71C5, and the computational methods were further used to identify the yet unknown molecular basis of ABA glycosylation as well as the regulatory role of persulfidation in this process. Our results suggest that the linker and the U-shaped loop are regulatory structural elements: the linker is associated with the binding of uridine diphosphate glucose (UPG) and the U-shaped loop is involved in binding both UPG and ABA.It was also found that it is through tuning the dynamics of the U-shaped loop that is accompanied by the movement of tyrosine (Y388) that the persulfidation of cysteine (C311) leads to the catalytic residue histidine (H16) being in place, preparing for the deprotonation of ABA, and then reorientates UPG and deprotonated ABA closer to the ‘Michaelis’ complex, facilitating the transfer of a glucose moiety. Ultimately, the persulfidation of UGT71C5 is in favor of ABA glycosylation. Our results provide insights into the molecular details of UGT71C5 recognizing substrates and insights concerning persulfidation as a possible mechanism for hydrogen sulfide (H_2_S) to modulate the content of ABA, which helps us understand how modulating ABA level strengthens plant tolerance.

## 1. Introduction

Abscisic acid (ABA), a very important phytohormone, regulates stress responses in plants [1]. The dynamic change in ABA level in tissues and cells is mainly reflected in two ways: one is when they are subjected to abiotic stresses, such as drought, where plants quickly accumulate ABA, which is followed by the activation of stress response genes; the other is when, in a normal environment, endosomatic ABA is restored to the base level, ensuring plant growth. Plants have evolved several regulation mechanisms related to ABA level, such as synthesis, degradation, metabolism, (de)-conjugation, and transport, to achieve a balance between growth and defense [2]. Pioneering studies have identified the de novo biosynthesis of ABA, which is involved in multistep enzymatic reactions. In short, C40 carotenoid zeaxanthin is converted to xanthoxin [3,4,5], and the direct C15 precursor of ABA is then oxidized to ABA after its export from plastids to cytosol [6,7,8]. The inactivation of ABA is achieved by irreversibly catabolizing to phaseic acid and then to dihydrophaseic acid or its epimer [9,10]. Additionally, the conjugation with uridine diphosphate (UDP)-glucose is a direct inactivation pathway for ABA, and ABA-glucosyl ester (ABA-GE) formation occurs as a consequence [11,12]. It has been discovered that, during physiological responses to environmental stimuli and stresses, the utilization of the inactive pool of ABA constituted by ABA-GE is an alternative source of ABA production [13,14], which involves single-step hydrolysis, which is more rapid than the multistep de novosynthesis.

The multigene family findings of glycosyltransferases (GTs) in plants point out that a large proportion of GTs directly affects cellular homeostasis through certain processes concerning conjugating hormones, secondary metabolites, and biotic and abiotic environmental toxins [15,16]. Accordingly, glycosylation catalyzed by GTs plays a vital role in orchestrating the bioactivity and metabolism of small molecules. In *Arabidopsis thaliana* (thale cress), which is commonly used as a model for plant genomics and metabolism [17], two *β*-glucosidase isoforms known as BGLU18 [13] and BGLU33 [14] were observed to catalyze the hydrolysis reaction of ABA-GE so as to release free ABA, and the glycosylation mediated by a UDP-glycosyltransferase (UGT) transformed free ABA into a conjugated form [11]. Besides the previously reported UGT71B6/7/8 [18], UGT71C5 was identified as a major ABA glycosyltransferase contributing to the glycosylation of ABA to ABA-GE through molecular–genetic, biochemical, and pharmacological approaches [19].

**Figure 1:**
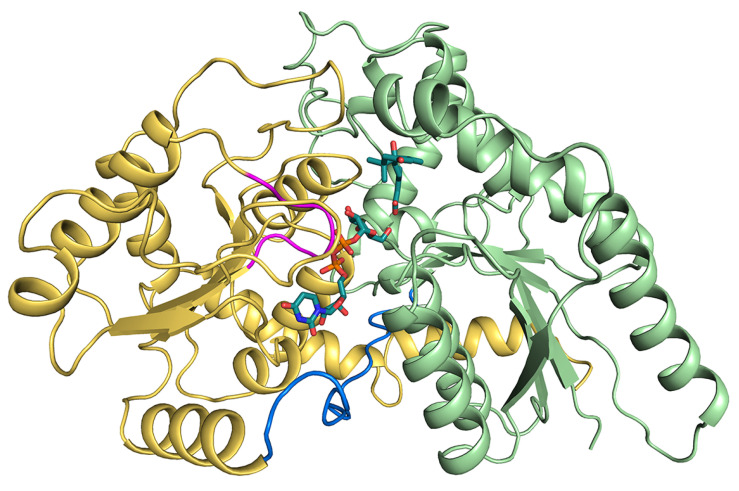
Structural information of UPG71C5 in complex with the substrates UPG and ABA. The N-terminal domain colored in lime green represents ABA binding; the C-terminal colored in yellow-orange represents UPG binding. The linker loop connecting the N- and C-terminal domains is shown in marine; the U-shaped loop constituting the UPG binding site is colored in magenta. The two substrates (UPG and ABA) are shown as sticks.

In the case of plants, UGTs catalyze a glycosidic bond formation using sugar donor containing a nucleoside phosphate, usually UDP-glucose (UPG) [20,21]. And, for nucleotide sugar-dependent enzymes, it is noted for its two structural folds: GT-A and GT-B [20]. Glycosyltransferase UGT71C5 adopts the GT-B fold, where the architecture consists of two *β*/*α*/*β* Rossmann-like domains, both featuring a central *β*-sheet. Two domains abutted by a flexible linker face each other, with the active sites positioned in the resulting clefts, the sizes of which vary, and the internal space in the N-terminal domain is provided for acceptor substrate binding, while the counterpart in the C-terminal domain is for the donor substrate [20,22] (Figure 1). Most plant UGTs present a common plant secondary product glycosyltransferase (PSPG) motif, and the conserved signature motif contains ~44 amino acids near the C-terminus [23,24,25], where the residues can identify the donor substrate [16]. In terms of UGT71C5, it contains a UGTPG motif similar to the PSPG motif at its C terminus [19]. In addition, GT-B-fold UGTs contain a conserved His–Asp catalytic pair in which the hydrogen bond between His and Asp is indispensable for the effective deprotonation of His through the charge relay system with a negatively charged Asp [26,27]. Among the UGTs of this type, glycosylation is likely to follow a typical inverting mechanism, that is, His would serve as a general base that deprotonates the hydroxyl group of acceptor substrates, and then anomeric C1 carbon on the glucose moiety is attacked by a nucleophilic oxyanion [22,28,29,30].

In plant systems, hydrogen sulfide (H_2_S), recognized as a signaling molecule, regulates the physiological–biochemical processes of plants’ performance [31,32]. The action of H_2_S is based on its ability to modify the thiol group (-SH) of cysteine residues in target proteins to form a persulfide group (-SSH), resulting in functional changes in protein structure, activity, or subcellular localizations [33,34]. This post-translational modification is called persulfidation [35], which has been demonstrated in plant critical processes, such as ABA-dependent stomatal movement [36,37,38]. The persulfidation of multiple components of the ABA signaling pathway has been recently characterized as a specific mechanism of action by which H_2_S is involved in ABA signaling regulation [39,40]. In contrast to the extensive research on the signaling transduction of ABA, few studies focus on the modulation of ABA level, particularly by (de)-conjugation. Interestingly, proteomic analysis detected persulfidated UGT71C5 in *Arabidopsis*, but without a specific persulfidated site [41].

We initially employed the modified biotin-switch technique (MBST) to identify the specific persulfidated site of UGT71C5, and then performed molecular dynamics (MD) simulations on a three-dimensional (3D) structure to determine the molecular basis of ABA conjugation and to ascertain whether the persulfidation of UGT71C5 is a potential mechanism by which H_2_S modulating ABA level. The persulfidation assays suggested that cysteine 311 in UGT71C5 is a NaHS-meditated persulfidation site. The simulations showed the activator role of the linker in initiating UGT71C5 rearrangement related to function and that the U-shaped loop serves as a regulatory structural element for accommodating substrate binding. Furthermore, our study found that the persulfidation of UGT71C5 contributes to the reorientation of UPG and ABA closer to the ‘Michaelis’ complex and leads to alternative release pathways for the acceptor substrate, which together make for ABA glycosylation. These analyses lay the foundation for the account of how conformational change is linked to the function of UGT71C5, and also present some insights into the molecular basis of UGT71C5 in recognizing substrate(s) as well as the played role of H_2_S regarding ABA level through UGT71C5 as a target of persulfidation. By intergrating in vitro persulfidation assays and in silico simulation experiments, this study investigates the persulfidation status of the UGT71C5 enzyme, a relatively unexplored area in the context of ABA homeostasis. Our study advances the understanding of the functional consequences related to UGT71C5 persulfidation through a structural analysis, revealing potential, new, regulatory network that influences ABA level. This methodological combination allows for establishing a direct link between UGT71C5 persulfidation and ABA metabolism, contributing original insights into plant hormone regulation.

## 2. Results

### 2.1. Identification of Persulfidation Sites in UGT71C5

**Figure 2:**
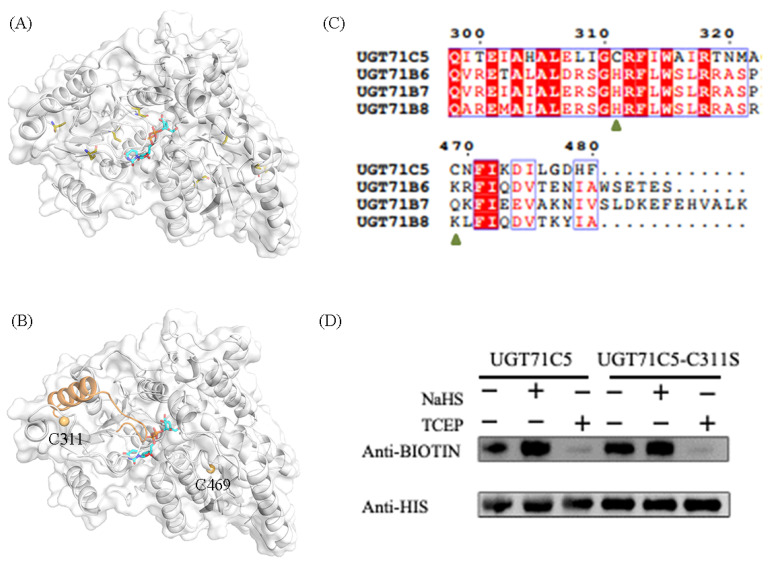
Persulfidation site and persulfidated protein assays in UGT71C5. (**A**) The spatial distribution of all cysteine residues in UGT71C5. The cysteine residues are shown as olive sticks. (**B**) The spatial distribution of the two predicted cysteine sites (C311 and C469) in UGT71C5. The two cysteine residues are presented as light-orange spheres and the UPG substrate is shown as a cyan stick. The *α*-helix structure connecting the situated loop of C311 and the U-shaped loop constituting the UPG binding site is colored in orange. (**C**) The sequence alignment of UGT71C5 and other UGTs inactivating ABA. (**D**) Quantification of the persulfidation level in UGT71C5 together with a C-to-S site-directed mutant. NaHS-induced persulfidation is detected by using the biotin-switch assay.

UGT71C5 contains seven cysteine residues (Figure 2A). We carried out the assessment of potential cysteine modification sites in UGT71C5 with the pCysMod server, a predictor developed based on deep learning (http://pcysmod.omicsbio.info/webserver.php (accessed on 10 July 2023)) [42]. The predictions made by the pCysMod server are C311 and C469 (Figure S1). The distribution of the two cysteine sites in UGT71C5 are illustrate in Figure 2B. It was noted that one *α*-helix structure connects the situated loop of C311 and the U-shaped loop constituting the UPG binding site; thus, it was expected that the persulfidation of C311 could induce a conformational effect and then influence the function of UGT71C5. We also performed a sequence alignment of UGT71C5 and other UGT enzymes, which transform free ABA into a conjugated form as well [18]. It was shown that site 311 is located in a relatively conserved region, while site 469 is characterized as a variable. Site 311 in UGT71C5 is a cysteine residue, and the equivalent is a histidine residue in other UGTs (Figure 2C).

Additionally, UGT71C5 was characterized as the major glucosyltransferase mediating ABA homeostasis [19], which underlines the significance of residue replacement in conserved region. To verify the persulfidation of UGT71C5 at C311, we applied a modified biotin-switch technique (MBST) for the persulfidation analysis [43]. The free cysteines of purified UGT71C5 were blocked with S-methymethane thiosulfonate (MMTS), the persulfidated cysteines were labeled with biotin, and the persulfidation levels were then quantitatively determined through a biotin antibody. As shown in Figure 2D, NaHS, a kind of H_2_S donor, induces the persulfidation of UGT71C5, and the application of tris (2-carboxyethyl) phosphine (TCEP) almost abolishes the persulfidation signal. Subsequently, we mutated cysteine 311 to serine in UGT71C5 and assessed the effect of NaHS on this mutated UGT71C5. It was observed that, compared to the UGT71C5 recombinant protein, NaHS treatment failed to trigger the increase in the persulfidation signal of C311S’s mutated UGT71C5. The data suggest that C311 is the persulfidated site in UGT71C5 in vitro.

Effective signaling through post-translational modification(s) requires stringent regulations. An enzyme’s catalytic activity is closely related to the precise structure of its active site. We then sought to determine whether persulfidation regulates the activity of UGT71C5 based on its structure with the help of computational technology. The protein UGT71C5 is considered for systems with the presence and absence of persulfidation as well as the protonation and deprotonation state for ABA into consideration. A total of six systems was examined, including substrate-free UGT71C5; UPG-bound UGT71C5; UGT71C5s in complex with UPG and ABA; and persulfidated UGT71C5s in complex with UPG and ABA. The resulting six systems that will be referenced in this paper are UGT71C5, UGT71C5-UPG, UGT71C5-UPG-ABA, UGT71C5-UPG-ABA^−^, UGT71C5-SSH-UPG-ABA, and UGT71C5-SSH-UPG-ABA^−^, with the superscript minus symbol on ABA indicating deprotonated ABA. We carried out three replicate explicit solvent molecular dynamics (MD) simulations for six forms of UGT71C5. Firstly, the C*α* root mean square deviations (RMSDs) of UGT71C5s were monitored during the simulations. As Figure S2 shows, the RMSDs of UGT71C5s are stable during the simulations with values smaller than 4 Å. And most analyses were performed for the last 150 ns equilibrated trajectories.

### 2.2. Structural Plasticity of UGT71C5 Associated with Substrate Binding and Persulfidation

**Figure 3:**
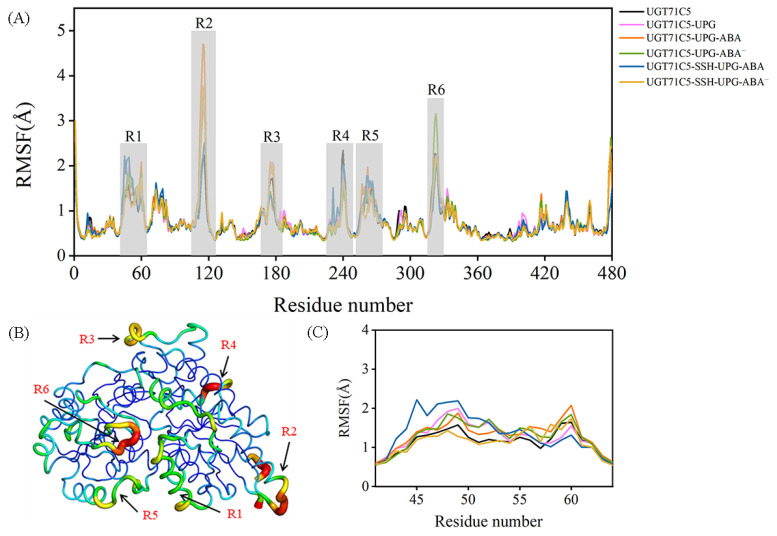
Root mean square fluctuation (RMSF) as a function of the residue number. (**A**) C*α* RMSFs of UGT71C5s. Six regions with even larger RMSF values are highlighted in light gray. (**B**) Sausage presentation of UGT71C5 system with the width and color (blue to red) related to the RMSF values. Six regions with higher flexibility. (**C**) The enlarged view of the RMSF profile of R1 region. Description of the systems: UGT71C5 refers to substrate-free UGT71C5; UGT71C5-UPG refers to UPG-bound UGT71C5; UGT71C5-UPG-ABA refers to UGT71C5 complexed with UPG and protonated ABA; UGT71C5-UPG-ABA^−^ refers to UGT71C5 complexed with UPG and deprotonated ABA; UGT71C5-SSH-UPG-ABA refers to persulfidated UGT71C5 complexed with UPG and protonated ABA; UGT71C5-SSHUPG-ABA^−^ refers to persulfidated UGT71C5 complexed with UPG and deprotonated ABA.

The backbone motility of UGT71C5 during the simulation process was measured in terms of the C*α* root mean square fluctuation (RMSF) values of each residue. The RMSF profile shows several fluctuated regions with RMSF values greater than 1.5 Å (Figure 3A). To visualize how the changes in RMSF values are distributed spatially, we display them according to a color scale for UGT71C5’s structure (Figure 3B). Among the fluctuated regions, R2, R3, and R4 belong to the protein’s exterior exposed to a solvent, which show a weaker interaction with the remaining structure; thus, they are liable to movement. Furthermore, varying degrees of flexibility were observed across all systems in the R1 region, associated with the helix structure at the lower side of the N-terminal domain, and in the R5 region, which functions as the linker between the N- and C-terminal domains. Relative to the loop structural element possessing an inherent flexibility, the mobility of the helix structure may be attributed to the surroundings, such as the side-chain movement of linker residues. We then focus on the local RMSF profile of the helix structure. As shown in Figure 3C, the binding of UPG alone or the binding of both UPG and ABA resulted in an increased RMSF value. In the UGT71C5-SSH-UPG-ABA system, several N-terminal residues had even larger values whereas the C-terminal ones showed the opposite trend, and in the UGT71C5-SSH-UPG-ABA^−^ system, only a slight increase was observed. Moreover, we noted an even larger RMSF value for R6 situated around the UPG binding site in the UGT71C5-UPG-ABA^−^ system relative to the remaining systems, suggesting a large positional change.

Then, we performed dynamical cross-correlation matrix (DCCM) analyses of the trajectories of the three regions (R1, R5, and R6) (Figure S3). The DCCM results show that a correlated motion exists between R1 and R5 in the UGT71C5 system. The concerted motion was bolstered in the UGT71C5-UPG system, and also a correlation within R1, R5, and R6 was observed. But, for systems with both UPG and ABA bound at their respective sites, the R1-R5 correlation decreased and the R1-R6 correlation turned into a negative correlation, as well as displaying an intra-regional correlation change. Taken together, the comparison of RMSFs together with DCCMs suggests that the three regions adjust their conformation accordingly in response to external stimulations, including substrate binding and persulfidation, and the differential changes among these systems underline the structural plasticity of UGT71C5.

### 2.3. Substrate Binding vs. Persulfidation: Effects on the Linker and the U-Shaped Loop

**Figure 4:**
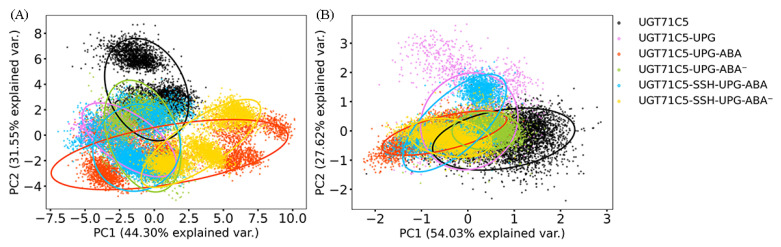
Rotated 2D principal component analysis (PCA) scatter plots for the first two principal components and 95% confidence ellipses: (**A**) the linker and (**B**) the U-shaped loop. The last 150 ns trajectories of all C*α* atoms are used as the dataset for PCAs with varimax rotations. PC1: first principal component; PC2: second principal component. The description of the systems is consistent with Figure 2.

Previous studies demonstrated a relationship between linker flexibility and protein function [44,45]. In order to determine the role of the linkers in UGT71C5, we carried out a principal component analysis (PCA) with a varimax rotation for the linker on the replicated simulations of all systems, which could help to characterize the essential dynamics. For the rotated PCA of the linker, the first two principal components (PC1 and PC2) (eigenvalues: 8.70 and 6.19, respectively) represented 75.85% of the variability, with PC1 explaining 44.30% of the total variance and PC2 explaining 31.55%. As shown in Figure 4A, the UGT71C5 system plays a considerable role in explaining the variation due to PC2 compared to PC1, and for the UGT71C5-UPG system, it demonstrates an increased contribution to the variability attributed to PC1 relative to the apo form. Meanwhile, the contribution to the variability along PC2 is notably reduced, suggesting the linkers in the UGT71C5 and UGT71C5-UPG systems likely differ in molecular dynamics, that is, the binding of UPG induces different behavior of the linker. In the bound form of UGT71C5 complexed with UPG and protonated ABA, it significantly contributes to the variability in PC1 and moderately in PC2, demonstrating a more pronounced presence in shaping the motion mode characterized by PC1 and a secondary involvement in the motion mode related to PC2. For the system where ABA deprotonated within the binding site, it explains a smaller proportion of the variability on PC1 and almost same proportion to PC2 compared with the UGT71C5-UPG-ABA system. The various behaviors of the linkers in the three cases, namely, the absence of ABA from the biding site and the presence of ABA in its protonated and deprotonated states, imply that there may be a structural element related to both substrate bindings. We then take the U-shaped loop into consideration as it constitutes UPG binding site and belongs to ABA surrounding in space. It is possible for the binding of ABA to exert influence over the behavior of U-shaped loop, further to UPG at binding site accompanied by alteration of the linker. Furthermore, the persulfidated site (C311) is located at a loop joining the *α*-helix and *β*-strand, where the *α*-helix is also a flank of the U-shaped loop, thus it is also possible for persulfidation to exert influence over the behavior the linker indirectly. The persulfidation indeed influences the dynamics of the linker as indicated by the change of contribution to the variability on both PC1 and PC2 in persulfidated systems compared to non-persulfidated counterparts. Together these results emphasized the plasticity of the linker as well as complicated dynamic behavior.

Next, we also performed a rotated PCA on the U-shaped loop to investigate its dynamics. In the case of the U-shaped loop, the eigenvalues associated with PC1 and PC2 are determined to be 0.71 and 0.36, respectively, and the first two components represent 81.65% of the total variance (PC1 = 54.03% and PC2 = 27.62%). As shown in Figure 4B, for the UGT71C5 system, it contributes to the substantial variability in PC1 and moderately to PC2, and for the UGT71C5-UPG system, it exhibits a heightened influence on the variability along PC2 while demonstrates a notably reduced impact on the variability captured by PC1, suggesting a significant alteration in the relative importance of motion modes between the two systems, and further to say, the binding of UPG influences the dynamics behavior of the U-shaped loop. Following the binding of protonated ABA, the behavior of the U-shaped loop is strongly aligned with the dominant motion mode represented by PC1 than by PC2, whereas upon the deprotonation of ABA, its behavior aligns less with the patterns captured by both PC1 and PC2. Together, these results highlight the diverse dynamics of the U-shaped loop in the UPG binding alone versus the binding of both UPG and ABA, suggesting that the U-shaped loop may function as a regulatory structural element responding to the binding of UPG and ABA.Moreover, concerning persulfidation, it was noted that, for the UGT71C5-SSH-UPG-ABA system, it makes less of a contribution to explaining the variability in PC1 while it displays a significantly increased impact on PC2 in comparison to the UGT71C5-UPG-ABA system, highlighting the shift in dynamics of the U-shaped loop with the changes in the motion characteristics induced by persulfidation. Then, for the UGT71C5-SSH-UPG-ABA^−^ system, although it plays minor role in explaining the variability in both PC1 and PC2 similar to the non-persulfidated counterpart, it was observed that it shifts to the left relative to the UGT71C5-UPG-ABA^−^ system along the PC1 axis, indicating a contrast in the direction of motion characterized by PC1 between the two systems. The displayed discrepancy between the persulfidated systems and non-persulfidated counterparts signifies that persulfidation influences the dynamics of the U-shaped loop. Furthermore, the effects of persulfidation could be manifested through the U-shaped loop.

### 2.4. Reduced Change in the Orientation of Deprotonated ABA in Persulfidated UGT71C5

**Figure 5:**
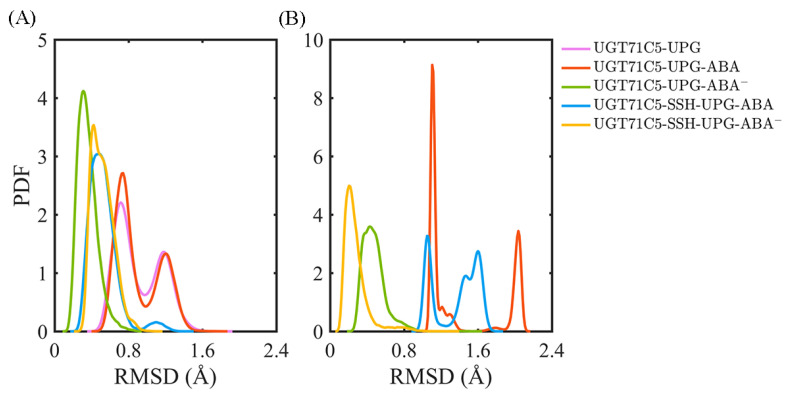
Analysis of the binding stability of two substrates for UGT71C5s. The probability distribution function (PDF) of RMSDs of substrate heavy atoms according to their average coordinates in different complex systems during the last 150 ns simulations. (**A**) Donor substrate UPG. (**B**) Acceptor substrate ABA. The description of the systems is consistent with Figure 2.

After characterizing the effects of substrate binding on the functional structural elements, we next analyzed the substrates themselves. In order to obtain information on positional fluctuations, the mobility of two substrates inside the binding site was monitored in the form of RMSD according to their average coordinates during the last 150 ns. For UPG (Figure 5A), we notice that the broadest bimodal probability distribution profile of RMSD appears in the UGT71C5-UPG system. For UGT71C5s complexed with both substrates, the RMSD distribution curve is one-humped and concentric in the UGT71C5-UPG-ABA^−^ system and is broader and bimodal in the UGT71C5-UPG-ABA system. Then, for persulfidated UGT71C5s complexed with both substrates, the overall RMSD curve is nearly identical, meaning that persulfidation indeed influences the motion of UPG, as we suggested previously. Relative to the UGT71C5-UPG-ABA system, the average of the RMSDs shifted to a lower value for the UGT71C5-SSH-UPG-ABA system, and relative to the UGT71C5-UPG-ABA^−^ system, a larger value is present for the UGT71C5-SSH-UPG-ABA^−^ system. The comparative results are consistent with the ones from the PCA analysis of the linker, which underline the relation of the linker to UPG. Furthermore, as for ABA (Figure 5B), the RMSD profiles show that ABA in its protonated form exhibits greater mobility, whereas deprotonated ABA fluctuates less. The key action in relative stabilization is the polar interaction between deprotonated ABA and the HIP16 residue. From the comparisons of the four UGT71C5 complexes bound by two substrates, it was found that ABA in both protonation and deprotonation states achieved even smaller RMSDs in the persulfidated systems than in their non-persulfidated counterparts, and the average RMSD for the ABA was the lowest in the case of the UGT71C5-SSH-UPG-ABA^−^ complex.

We further took a deep dive into the fluctuation of two substrates through RMSD calcu-lation applied to the decomposed fragments (glycosyl moiety, phosphate moiety, and uridine moiety) from the UPG molecule (Figure S4A) and dihedral angle measurements of the rotatable bonds for ABA (Figure S4B). In further detail (Figure S5), the RMSDs show that the motility of UPG in the UGT71C5-UPG system mainly comes from the atom fluctuations in the glycosyl moiety and uridine moiety. Upon either protonated or deprotonated ABA binding to acceptor binding site, the glycosyl moiety exhibits stably as the RMSD profiles shown in the UGT71C5-UPG-ABA and UGT71C5-UPG-ABA^−^ systems. Additionally, concerning the atoms of the phosphate moiety and uridine moiety, this suggests that the two fragments contribute to the fluctuation in UPG in the UGT71C5-UPG-ABA system. Furthermore, in the case of persulfidated UGT71C5s, the glycosyl moiety presents larger positional fluctuations inside the binding site relative to in its non-persulfidated counterparts, which together with the phosphate moiety, is the driver of the positional fluctuation in UPG. These results reveal that the binding of ABA in both states can help stabilize the glycosyl moiety, but the persulfidation of UGT71C5 further triggers its positional fluctuation. It was also indicated that UPG exhibits high stability in the UGT71C5-UPG-ABA^−^ system compared to other systems.

The joint dihedral angle distributions (Figure S6) show that protonated ABA adopts various conformations inside the binding site, and protonated ABA in the persulfidated system behaves differently to its non-persulfidated counterpart. However, the mobility for deprotonated ABA was reduced, most notably for the ABA in the UGT71C5-SSH-UPG-ABA^−^ system. For ABA in protonation and deprotonation conditions, a key difference is the state of the carboxyl group of ABA, its un-ionized form corresponding to protonated ABA and its ionized form to deprotonated ABA. Deprotonated ABA can form a polar interaction with residue H16, which provides certain stability for ABA inside its binding site. Moreover, the six-membered ring of ABA is also a movement factor, of which the dynamic performance can be characterized by dihedral angle 1 (D1). Following the comparison of the corresponding dihedral angle data for the four ABA-bound complexes, it was noted that D1 at around zero degrees was sampled, excluding the case of deprotonated ABA binding to persulfidated UGT71C5, suggesting that the orientation of the six-membered ring differed in the UGT71C5-SSH-UPG-ABA^−^ system compared to other systems. For the two systems featuring polar interactions, the discrepancy of RMSD values for ABA mainly arises from the motion of the six-membered ring. A structure superposition (Figure S7) helps display the result that the different behaviors of the U-shaped loop lead to the conformational heterogeneity of its residue M291. M291 is hydrophobic and the six-membered ring of ABA together with methyl substituents is also a non-polar fragment. Thus, the conformational change in the M291 side-chain may perturb the ring fragment of ABA in view of the close spatial location.

### 2.5. A Dynamic Microenvironment for UPG Binding

**Figure 6:**
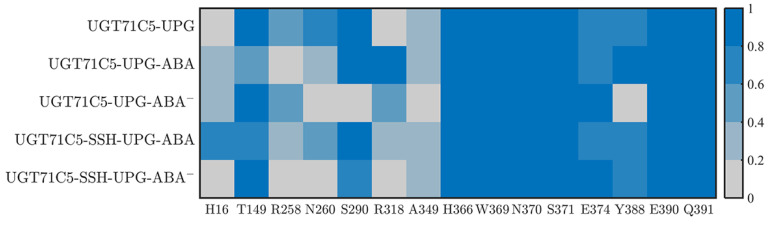
The hydrogen bonding interactions between UPG and UGT71C5. The color indicates the proportion of hydrogen bonds during the simulation. The description of the systems is consistent with Figure 2.

The previous results demonstrate the behavioral variability in UPG inside binding site in various UGT71C5s. The binding stability of the substrate could be linked to residues surrounding the structure. Firstly, as shown in the starting structure (Figure S8A), among the surrounding residues, several of the residues (H366, W369, N370, S371, E374, Y388, E390, and Q391) of the conserved PSPG motif form hydrogen bonds with UPG. Moreover, two residues, S290 and A349, located outside the PSPG motif, also participate in binding UPG through hydrogen bonds. We next identified the dynamic performance of these hydrogen bond interactions between UPG and surrounding residues for more detailed information. Since the UPG attached to the pocket is in a stable position in the UGT71C5-UPG-ABA^−^ system, its representative structure obtained by cluster analysis is checked to analyze new hydrogen bond connections (Figure S8B). As shown in the hydrogen bond interaction map (Figure 6), for the UGT71C5-UPG system, E374 and Y388 exhibit reduced hydrogen bond interactions with UPG than the other residues in the PSPG motif. Specifically, E374 forms interaction with the atoms of uridine moiety, thus certain damage probably because of positional deviation of the uridine moiety, and Y388 forms interaction with the phosphate moiety, but large mobility uncharacterized in the previous RMSD calculation on phosphate moiety, thus the certain damage is mainly attributed to the movement of Y388 itself. Additionally, there are available hydrogen bonds between UPG and the two residues (R258 and N260) within the linker. Initially, the two residues are positioned away from UPG, particularly R258. The hydrogen bonds involve atoms from the uridine moiety of UPG, and the movement of the residue side chain plays a significant role in establishing these interactions. The uridine moiety of UPG may also undergo displacement regarding the initial position, showing that the linker is linked to UPG binding. Then, for the UGT71C5-UPG-ABA system, the proportion of hydrogen bonds involving linker residues is much less than in the UGT71C5-UPG system, but another hydrogen bond between UPG and R318 is observed, meaning that this hydrogen bond concerning the uridine moiety is always available during simulations. According to the results of the RMSD calculated for the uridine moiety, the even larger magnitude of motion exhibited in the UGT71C5-UPG-ABA system could be related to residue R318. In the UGT71C5-UPG-ABA^−^ system, notable differences on proportion of hydrogen bond formation exist between UPG and S290/Y388. The two initial interactions are destroyed. S290 is situated on the U-shaped loop and Y388 is situated close to the U-shaped loop; thus, this shows that the U-shaped loop is in a distinct spatial position in the UGT71C5-UPG-ABA^−^ system. For the persulfidated systems, the hydrogen bonds involving residues R258, N260, and R318 are available in the UGT71C5-SSH-UPG-ABA system, albeit in a small proportion, but are not in the UGT71C5-SSH-UPG-ABA^−^ system. And, the hydrogen bond proportion of S290 reduces in the UGT71C5-SSH-UPG-ABA^−^ system. In summary, these results underline the dynamic performance for the binding site of UPG, and both the binding of ABA and persulfidation induce the rearrangement of binding site residues surrounding UPG.

### 2.6. Alternative Conformations for Long Side-Chain Residues Highlight the Fluctuation in Donor Substrates

In the starting UGT71C5 structure complexed with UPG (Figure S9), in the local hydrogen bond network, R318 forms side chain–main chain hydrogen bonds with S290 and M321, and the side chain of S290 forms a hydrogen bond with the sugar donor, contributing to maintain a particular conformation, and an additional interaction of π-stacking between UPG and W348 occurs. Typically, at the residue level, the bond rotation and rotation angle are vital in creating notable conformational changes. It is expected that the following analysis at the residue level could provide some insights into the detected discrepancies in various UGT71C5 structures. Considering that long side-chain residues have more alternative conformations and are liable to perturb other residues, once the conformational changes occur on their own, we primarily focus on four residues (R258, M291, R318, and W348). Upon the alignment of trajectories relative to the initial structure to eliminate translational rotations, we obtained the positional changes in these residues in three dimensions during the simulations. The distributions of the centers of sidechain heavy atoms reveal that the sampling locations for each of the four residues do not perfectly overlap in all systems and, in some cases, are even scattered. This observation reflects the dynamic nature of these residues and their conformational heterogeneity (Figure S10).

**Figure 7:**
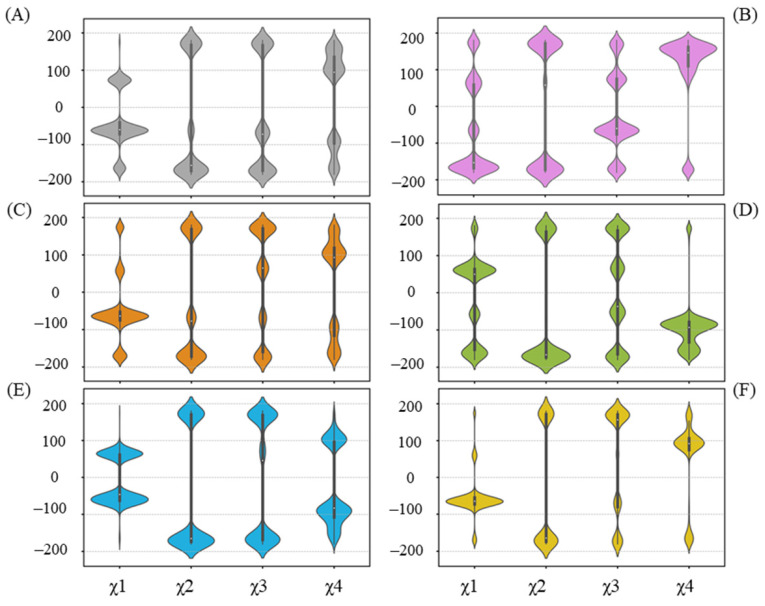
Distribution of side-chain chi (χ) angles of R258 in each system. (**A**) UGT71C5 system; (**B**) UGT71C5-UPG system; (**C**) UGT71C5-UPG-ABA system; (**D**) UGT71C5-UPG-ABA^−^ system; (**E**) UGT71C5-SSH-UPG-ABA system; (**F**) UGT71C5-SSH-UPG-ABA^−^ system. The description of the systems is consistent with Figure 2.

Residues that have undergone side-chain conformational changes are typically characterized by changes in their chi (χ) angles. In terms of R258 (Figure 7), related to the UGT71C5 system, a reorientation in the UGT71C5-UPG system occurs, mainly manifested by χ^1^ and χ^4^ changes, making it capable of forming a hydrogen bond with UPG. The hydrogen binding interaction between UPG and R258 is also present in the UGT71C5-UPG-ABA^−^ and UGT71C5-SSH-UPG-ABA systems, as shown in Figure 6. Then, observing the χ angle data shows that the distributions of χ^1^ and χ^4^ in the UGT71C5-UPG-ABA^−^ system are located in the opposite region compared to the UGT71C5 system, indicating that R258 takes on distinct rotamer, and in the UGT71C5-SSH-UPG-ABA system, the two χ angles display a partial agreement as in UGT71C5-UPG-ABA^−^ system, which explains the relatively small proportion of hydrogen bond formation. Although the interaction is not present in the remaining complexes, the χ angles of R258 appear to present multiple distributions. Obviously, the conformational isomers of R258 destabilize the linker loop that it belongs to. A result arising from the alteration in the linker isthe motion of N260, which is determined by the order parameter calculation (Figure S11). W348, a spatially adjacent amino acid of N260 (Figure S12A), can be influenced by N260’s motion, which in turn affects the movement of R318. The previously determined 3D coordinates data of R318 and W348 support their observed mobility.

**Figure 8:**
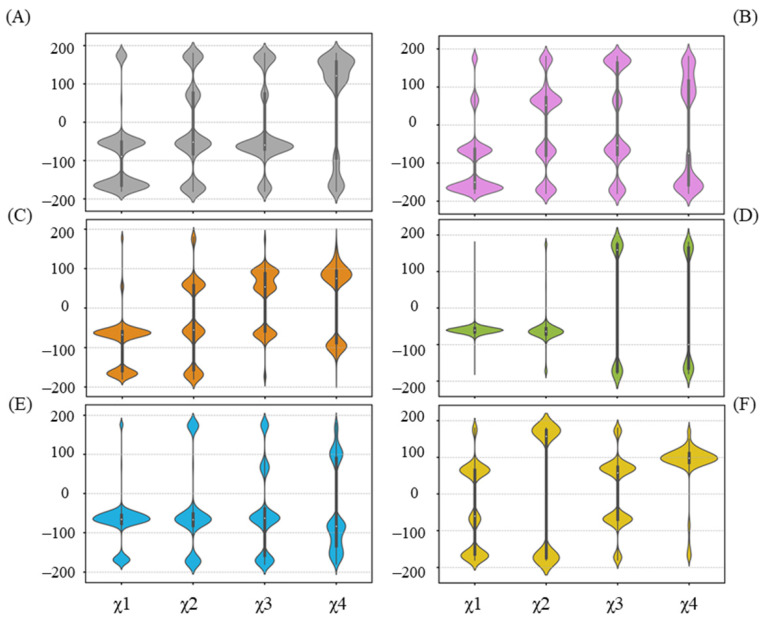
Distribution of side-chain chi (χ) angles of R318 in each system. (**A**) UGT71C5 system; (**B**) UGT71C5-UPG system; (**C**) UGT71C5-UPG-ABA system; (**D**) UGT71C5-UPG-ABA^−^ system; (**E**) UGT71C5-SSH-UPG-ABA system; (**F**) UGT71C5-SSH-UPG-ABA^−^ system. The description of the systems is consistent with Figure 2.

The hydrogen bond between UPG and R318 is not present in all complex systems, as depicted in Figure 6. Furthermore, in cases where the hydrogen bond forms, it occurs in varying proportions. We thereby examined the χ angle data of R318 (Figure 8). Overall, the side chain of R318 exhibits high conformational flexibility, as indicated by the diverse distributions that vary among each system. As noted previously, R318 gains a larger proportion of hydrogen bond formation with UPG in the simulations of the UGT71C5-UPG-ABA system, and the corresponding χ angle data of R318 highlight its conformational variability. To maintain hydrogen bond formation, UPG likewise experiences positional fluctuations, which accounts for the larger RMSD value of the uridine moiety. However, in the UGT71C5-UPG-ABA^−^ system, R318 is stable and takes on a specific conformational isomer, as the distribution of the χ angle is distinct to other systems. Combined with the previous RMSF analysis, which showed the largest amplitude of R6’s motion in the UGT71C5-UPG-ABA^−^ system, it is evident that this larger motion allows R318, a residue of R6, to extend its side chain.. Moreover, according to the hydrogen bonding interaction of R318 with other residues (Figure S12B), it was noted that the original hydrogen bond with S290 was broken during the simulations, a robust bond with F288 was formed, and the bond with G289 was available as well in the UGT71C5-UPG-ABA^−^ system. The distinct conformational isomer presented by R318 re-established its association with the U-shaped loop, leading to different orientations of the U-shaped loop, further disrupting hydrogen bonding between S290 and UPG. The stable behavior of R318, combined with the minimal conformational space sampled by the U-shaped loop, contributes to the stable binding of UPG in the UGT71C5-UPG-ABA^−^ system.

**Figure 9:**
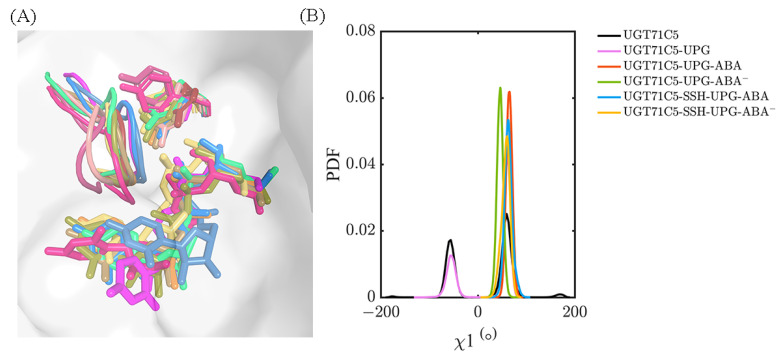
(**A**) The conformations of the U-shaped loops in representative structures selected based on clustering analysis. Salmon, warm pink, and firebrick correspond to UGT71C5 system; magenta and hot pink correspond to UGT71C5-UPG system; orange and yellow-orange correspond to UGT71C5-UPG-ABA system; lime green corresponds to UGT71C5-UPG-ABA^−^ system; marine and sky blue represent the UGT71C5-SSH-UPG-ABA system; sand and deep olive correspond to UGT71C5-SSH-UPG-ABA^−^ system. UPG and Y388 are shown as colored sticks, with their colors matching those of the corresponding U-shaped loops. (**B**) The probability distribution function (PDF) of the χ^1^ angle of Y388. The description of the systems is consistent with Figure 2.

Then, in regard to the two persulfidated systems, the original hydrogen bond between R318 and S290 is reduced in the UGT71C5-SSH-UPG-ABA system and fails to form in the UGT71C5-SSH-UPG-ABA^−^ system. Viewing the representative structure of each system could allow us to obtain more information. As shown in the representative structures (Figure S13), R318 adopts various conformational isomers in each system indicated by its side-chain orientation, and S290 takes on different orientations in the persulfidated systems relative to the non-persulfidated counterparts, which is indicative of the U-shaped loop with changes across the systems. Through the superposition of representative conformations, we found that the U-shaped loop with its position in the UGT71C5-UPG-ABA^−^ system as a reference shows displacements in both the left and right directions, together with the downward direction. The motion of the U-shaped loop would certainly render the fluctuation of UPG and cause spatial changes in the surroundings, such as enabling Y388 to move in a flip mode or undergo slight positional fluctuations. We then characterized the mobility of Y388 through its χ^1^ angle data. As shown in Figure 9, the bimodal probability distribution profile appears both in the UGT71C5 and UGT71C5-UPG systems, and the distribution is restricted to a unimodal shape after the binding of protonated or deprotonated ABA. The average value of Y388 ‘sχ^1^ angle is 64.80 ± 6.45° in the UGT71C5-UPG-ABA system, whereas it is 45.80 ± 6.45° in the UGT71C5-UPG-ABA^−^ system, deviating from the starting value of 65.60°, which is just one of the reasons for the broken hydrogen bond between Y388 and UPG. For the two persulfidated systems, the average value is 63.34 ± 8.44° in the UGT71C5-SSH-UPG-ABA system and 57.41 ± 8.82° in the UGT71C5-SSH-UPG-ABA^−^ system. It is worth noting that an even broader distribution range is exhibited in persulfidated systems compared to their non-persulfidated counterparts, which helps explain the even larger RMSD values of the glycosyl moieties observed in two persulfidated systems. Together, the movements of the U-shaped loop itself and the Y388 residue eventually affect the behavior of the UPG attached to the donor binding site. In brief, these results reveal that the binding site of UPG undergoes a rearrangement by residue side-chain conformational changes. As several key residues display different positions across the systems, UPG accordingly behaves differently.

### 2.7. Preferable Configuration Condition for Glycosylating ABA Induced by Persulfidation

The microenvironment surrounding substrates varies in various UGT71C5 structures. Undoubtedly, glycosylation requires proper spatial arrangement, and glycosylation activity is related to the spatial orientations of donor and acceptor substrates and critical residues as well. As for UGT78K6, the distance between the Nε2 atom of H17 and the anomeric C1 carbon of glucose is 5.2 Å, and the 3-OH group of its acceptor substrate (kaempferol) to be glucosylated forms a hydrogen bond with the catalytic residue H17, with a distance of 2.5 Å [30]. Regarding *Pa*GT3, the 3-OH group of kaempferol is at distance of 2.9 Å from catalytic H20 and 3.8 Å from the C1 atom in the glycosyl moiety in molecule B of *Pa*GT3, and this binding orientation of kaempferol is likely to be a close representation of the ‘Michaelis’ complex [27]. As far as *Vv*GT1 is concerned, the attacking O3 hydroxyl of the flavonoid lies 2.7 Å from H20, and the geometry is exquisitely poised ‘in-line’, with an O3_kaempferol_-C1_UDP-Glc_-O1_UDP-Glc_ angle of 160°, as expected for the ‘Michaelis’ complex in a single displacement mechanism [24].

**Figure 10:**
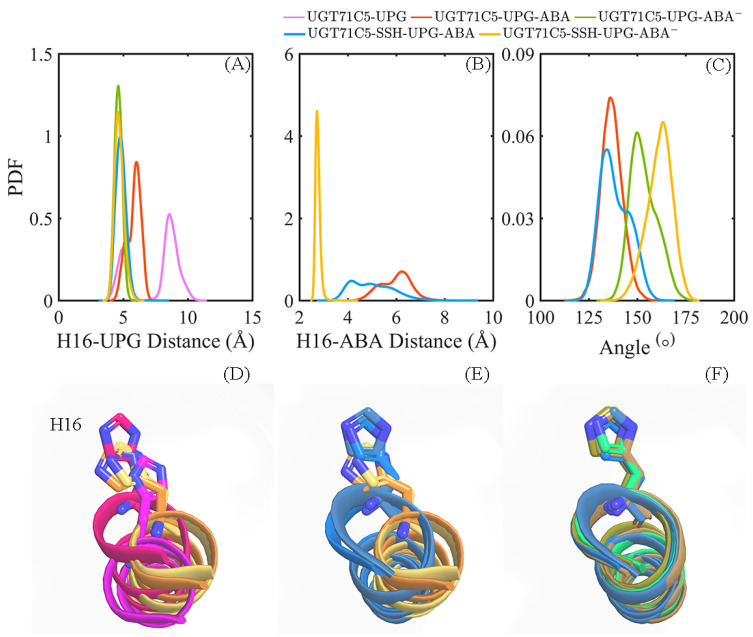
Information related to ABA glucosylation. The probability distribution function (PDF) of the distances between the Nε2 atom of H16 and anomeric C1’ atom of UPG (**A**), and the O atom on the hydroxyl group of ABA (**B**). (**C**) The PDF of the O_ABA_-C1_UPG_-O1_UPG_ angle. (**D**–**F**) Alignments of the *α*-helix structure containing H16, based on representative structures. (**D**) Alignment of UGT71C5-UPG structures in magenta and hot pink, and UGT71C5-UPG-ABA structures in orange and yellow-orange. (**E**) Alignment of UGT71C5-UPG-ABA structures in orange and yellow-orange, and the UGT71C5-SSH-UPG-ABA structures in marine and sky blue. (**F**) Alignment of UGT71C5-UPG-ABA^−^ structure in lime green, UGT71C5-SSH-UPG-ABA structures in marine and sky blue, and UGT71C5-SSH-UPG-ABA^−^ structures in sand and deep olive.

According to the mentioned information, we further performed calculations on the distance of the Nε2 atom of H16 from the anomeric C1’ carbon atom of UPG in UGT71C5 as well as the reactive hydroxyl group of ABA and the angle of its *Vv*GT1 counterpart. As shown in the distance profiles (Figure 10A), for the UGT71C5-UPG system, the distance of the Nε2-C1’ atom pair has the broadest bimodal probability distribution profile, with an average value of 7.49 ± 1.89 Å, and when ABA attaches to UGT71C5, the Nε2-C1’ distance appears as a unimodal distribution, with an average value of 5.82 ± 0.54 Å in the UGT71C5-UPG-ABA system and 4.62 ± 0.29 Å in the UGT71C5-UPG-ABA^−^ system. Then for the two persulfidated systems, the distribution profile for the distance of Nε2-C1’ is also unimodal, with an average value of 4.82 ± 0.39 Å in the UGT71C5-SSH-UPG-ABA system and 4.70 ± 0.36 Å in the UGT71C5-SSH-UPG-ABA^−^ system. As shown in Figure 10B, for the two cases of protonated ABA binding to its site, the distance of the Nε2-O atom pair displays a broader distribution, with the average value shifted left in the UGT71C5-SSH-UPG-ABA system relative to the UGT71C5-UPG-ABA one. And, for other two deprotonated ABAs attached to the binding site, the distribution profile for Nε2-O’s distance is concentric, with an average value of 2.81 ± 0.13 Å for the UGT71C5-UPG-ABA^−^ system and 2.76 ± 0.10 Å for the UGT71C5-SSH-UPG-ABA^−^ system. This emphasizes the importance of the deprotonation of ABA by catalytic H16 for glycosylation. In terms of the angle proxy (Figure 10C), an average angle is around 130° for the two protonated ABA-bound UGT71C5 structures, but a small bump on the right side of the curve in the UGT71C5-SSH-UPG-ABA system is present, meaning that the distribution of the angle data is slightly heavier on the right side. Furthermore, the average value is 153.37 ± 6.98° for the UGT71C5-UPG-ABA^−^ system and 161.46 ± 6.50° for the UGT71C5-SSH-UPG-ABA^−^ one. There is an indication that the mutual orientation between UPG and deprotonated ABA in persulfidated UGT71C5 is comparable to the one in the *Vv*GT1 complex structure, close to the ‘Michaelis’ complex required for glycosylation.

As a typical inverting mechanism, histidine would serve as a general base that deprotonates the hydroxyl group of acceptor substrates, and then anomeric C1 carbon on glucose is attacked by a nucleophilic oxyanion [20]. This mechanism highlights that histidine plays an essential role. Thus, we carry out an alignment on the *α*-helix structure, which contains H16. From the alignments on representative structures (Figure 10D–F), we observed that *α*-helix’s structure does not assume the same position in its two representative structures in the UGT71C5-UPG system, supporting the bimodal distribution of the distance of Nε2-C1’. For the two ternary complexes with protonated ABA attached to the acceptor binding site, the *α*-helix structure has same position in each system’s representative structures, but not when comparing the two systems. Interestingly, the position of the *α*-helix structure in UGT71C5-SSH-UPG-ABA is comparable to that in the two systems with deprotonated ABA binding. This explains that both the distance profiles for Nε2-C1’ and Nε2-O shift left in the UGT71C5-SSH-UPG-ABA system relative to the UGT71C5-UPG-ABA system. Overall, for the protonated ABA, the appropriate position of H16 is particularly critical as ABA needs to be deprotonated by H16, and for deprotonated ABA, the right value for the O_ABA_-C1_UPG_-O1_UPG_ angle is the key to transferring the glucose moiety. For the UGT71C5 complexed with UPG and protonated ABA, the persulfidationresults in H16 in preferable position, and for the UGT71C5 complexed with UPG and deprotonated ABA, persulfidation leads to the orientation between the sugar donor and accpetor closer to the ‘Michaelis’ configuration. In other words, the persulfidation of UGT71C5 contributes to ABA glycosylation.

### 2.8. Better Performance of Acceptor Substrate Dissociation Exhibited in Persulfidated UGT71C5

**Figure 11:**
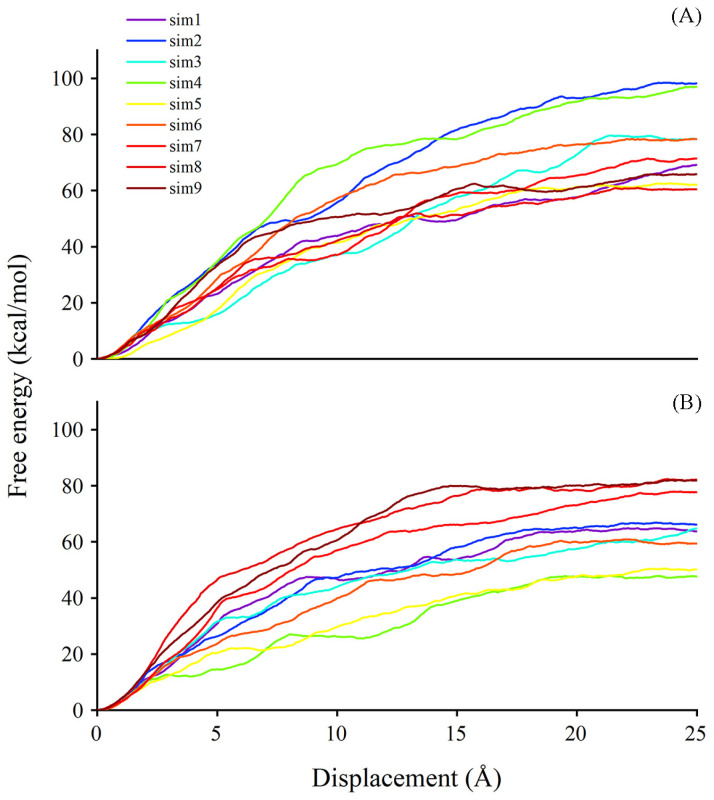
The free energy profile for ABA dissociation as a function of the distance of the deprotonated ABA center of mass from the binding site during SMD simulations. (**A**) UGT71C5-UPG-ABA^−^ system and (**B**) UGT71C5-SSH-UPG-ABA^−^ system.

It is known that, besides degradation, ABA can be stored in a glycosylated form, named ABA-GE, by the action of UGTs. The former analyses focused on substrate binding. We next employed the steered molecular dynamics (SMD) technique to obtain information concerning substrate dissociation due to the timely release of sugar acceptor facilitating the subsequent cycle of glycosylation. Using the SMD method, we could achieve a sense of the possible pathways for ligands exiting the binding site and the estimated free energy in corresponding pathways. The last frame from the conventional molecular dynamics (CMD) simulation trajectory was selected as an initial configuration for the SMD simulation. The plotting of free energy profiles suggests that the work conducted for ABA out of the binding site ranges from 60 to 100 kcal/mol in the UGT71C5-UPG-ABA^−^ system, and the range is from 45 to 85 kcal/mol in the UGT71C5-SSH-UPG-ABA^−^ system (Figure 11). The comparison of egress routes of ABA from UGT71C5 shows that the conformation of the disordered loop (R6 mentioned previously) in proximity to the entry site for the donor is somehow related to acceptor dissociation. The ‘portal region,’ formed by its outward movement away from the donor binding pocket, provides an alternative exit path for the dissociation of the acceptor substrate, aside from the one involving only the N-terminal domain. This alternative portal is highly preferable for the egress of ABA from the binding site, especially once the disordered loop achieves a certain level of openness. The opening of the ‘portal region’ is intrinsically connected to the dissociation process.

For the dissociation of ABA in the UGT71C5-UPG-ABA^−^ system, we observed one path involving the ‘portal region’ in the nine trials performed on ABA (Figure S14A–C). Three different egress routes are found for ABA exiting the binding pocket in the UGT71C5-SSH-UPG-ABA^−^ system, termed paths 1, 2, and 3. Path 1, situated in the lower region, is encased by the linker and a helix structure of the N-terminal domain (Figure S14D). Path 2 is accessible through the ‘portal region’, which is created by the outward shift of the disordered loop (Figure S14E). Path 3 is formed at the edge loop helix exposed to the solvent region and presented around the acceptor substrate binding cavity (Figure S14F). Although the ‘portal region’ was limited in the UGT71C5-UPG-ABA^−^ system, it was still visited when ABA exited the cavity, with a larger value of dissociation free energy as a consequence. For the persulfidated system, the release of ABA presented the lowest value of free energy through path 2 due to the larger accessible ‘portal region’, and relatively greater values appeared in the trials through path 3. Path 1 is not a conserved egress route, of which the formation is associated with the dynamical and conformational changes in related structural elements upon substrate binding. In a word, the plasticity of the disordered loop possibly plays a role in the dissociation of acceptor substrate, and the overall reduction in the amount of work required and the increased availability of alternative paths for sugar acceptors exiting the persulfidated UGT71C5 system may enhance the efficiency of receptor release, facilitating subsequent glycosylation processes..

## 3. Discussion

Plant glucosyltransferases (GTs) have attracted considerable interest for their diverse roles in natural product metabolism [46]. Glycosylation carried out by GT enzymes is a key mechanism that affects the activity of plant hormones.. Several UGTs from *Arabidopsis* have been identified to control ABA homeostasis, including UGT71B6 and its two closely related homologs, UGT71B7 and UGT71B8 [18], and UGT71C5. Multiple experimental studies have been performed with ABA glucosyltransferases, and through the comparisons of their roles in ABA-related phenotypes, UGT71C5 was characterized as a major ABA glucosyltransferase in *Arabidopsis* [19]. Our study is expected to describe the molecular basis of UGT71C5 sensing substrates (UPG and ABA) by studying the three-dimensional structure of UGT71C5 in various forms and provide insights into persulfidation as a possible mechanism for hydrogen sulfide (H_2_S) to modulate the content of ABA with UGT71C5 as a target.

**Figure 12:**
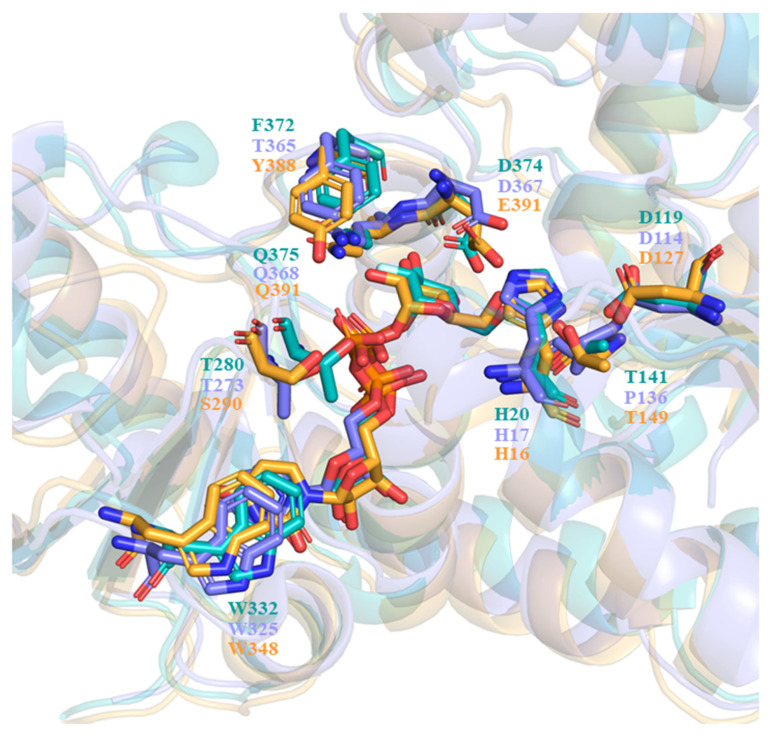
Comparison of the sugar donor binding schemes. *Vv*GT1 structure bound with UDP is shown in slate, the UGT78K6 structure bound with UDP-2FGlc is shown in teal, and the UGT71C5 structure bound with UPG (our study) is shown in bright orange. All donors (UDP, UDP-2FGlc, and UPG) and conserved residues are shown as sticks, with their colors matching those of the corresponsing protein structures.

In describing the substrate recognition of UGT71C5, the structure of donor and acceptor binding sites should be noted. The structure of the UPG-binding site in UGT71C5 is compared to that of other enzymes with similar enzymatic functions, like the flavonoid UGT *Vv*GT1 from *V. vinifera* (PDB ID: 2c1z [28]) and anthocyanidin UGT78K6 from *Clitoria ternatea* (PDB ID: 4whm [30]). The comparison of the donor binding schemes is shown in Figure 12. It was found that two residues interacting with glycosyl moieties were invariant between *Vv*GT1 and UGT78K6, that is, D374 and Q375 in *Vv*GT1 were conserved as D367 and Q368 in UGT78K6. In the case of UGT71C5, Q391 is equivalent to its counterparts, but E390 instead of D374 in *Vv*GT1 and D367 in UGT78K6 participates in the recognition of the glycosyl moiety. Additionally, another residue, T141, in *Vv*GT1 that interacts with the glycosyl moiety is replaced by P137 in UGT78K6, whereas it is retained as T149 in UGT71C5. Therefore, as it were, the position of the glycosyl moiety would be basically same in the functionally similar UGTs.

**Figure 13:**
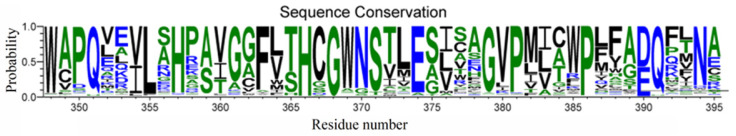
A consensus signature motif known as PSPG in most plant glucosyltransferases. Residues are numbered by the reference to UGT71C5. The sequence alignment obtained from webserver (https://gremlin.bakerlab.org/ (accessed on 5 January 2024)) and the generated figure using webserver (https://weblogo.threeplusone.com/ (accessed on 5 January 2024)).

Furthermore, most plant GTs present a consensus signature motif known as PSPG in the C-terminal domain, which defines the pocket environment for sugar donor substrates [20], but with respect to the specific residue composition, there may be some variations in plant enzymes (Figure 13). Indeed, it was noted that one residue of the PSPG motif, F372 in *Vv*GT1 and F365 in UGT78K6, was replaced by Y388 in UGT71C5 with the corresponding positions occupied. Relative to the hydrophobic side chain of phenylalanine, the substitution by tyrosine introduces a polar hydroxyl group, and this group can interact with the phosphate group and glycosyl moiety of UPG in the form of a hydrogen bond because of the sufficiently close location. It is worth noting that focusing on the dynamic change in the UGT71C5 protein rather than the static single-frame structure allows us to obtain more detailed information of the binding pocket. The side-chain χ angle data of Y388 show that it is also associated with ABA binding, as well as UPG, suggesting that Y388 might serve as a functional residue of UGT71C5. Of course, in addition to hydrogen bonding interactions, the sugar donor substrate also interacts with the residue in the UGT signature PSPG motif in another specific form, which manifests as π-stacking between the uracil base and tryptophan. Tryptophan is conserved as W332 in *Vv*GT1 and W325 in UGT78K6, as well as W348 in UGT71C5. However, the π-stacking interaction was broken during the simulations of UGT71C5, which resulted from the flip motion of W348. Likewise, we also analyzed that the flip motion of W348 resulted from the disturbance caused by the spatial positional change in the linker connecting the N- and C-terminal domains of UGT71C5. Although many PSPG motif residues involve the recognition of a sugar donor substrate, our results reveal that the linker part is also closely related to sugar donor substrate binding. Outside of the PSPG motif, a loop region is also located at the sugar donor binding site. The loop containing residues ranging from T273 to P277 in UGT78K6 was recognized to be flexible due to the average temperature factor, which is 51.5 Å in the case of an unliganded form, whereas this is significantly reduced to 22.7 Å with a binding donor through T273 interacting with the donor in a hydrogen bonding form [30]. The counterpart in UGT71C5 is a U-shaped loop comprising residues from S290 to F294, and S290 in UGT71C5 corresponds to T273 in UGT78K6. In the case of empty UGT71C5, the U-shaped loop exhibited its flexibility. The hydrogen-bonding interaction involved in S290 is available after UPG bound at the donor-binding site (Figure 6). Unlike in UGT78K6, the flexibility of the U-shaped loop in UGT71C5 was significantly restricted upon the binding of both UPG and ABA (Figure 4). Thus, this shows that the U-shaped loop plays the important function of accommodating the binding of substrates.

In regard to the sugar acceptor binding site, the domain is generally less conserved in order to accommodate diverse acceptors [47]. However, several key charged residues in the acceptor binding pocket are invariant among the majority of GT-B enzymes. They act as the general base triggering the deprotonation of the acceptor. Both histidine and aspartic acid can be used as a base [48]. For a case in which histidine is the base, it was proposed that histidine formed an acceptor-H-D triad, where the aspartate residue balanced the charge on the histidine after proton abstraction [49]. Some mutation experiments highlight the vital role of these residues in the nucleophilic attack on the acceptor, like the mutations of H19A and D117A, which resulted in the complete loss of *At*UGT72B1 activity [50]; the substitution of H22 and D121 with alanine in UGT71G1 resulted in inactive variants [49]; and the mutation of H20A aborted the catalytic activity of *Vv*GT1 [28]. In the case of UGT71C5, the equivalent catalytic residues are H16 and D127. The charged and uncharged states for ABA were simulated separately in our study so that we could examine the significance of histidine in UGT71C5. Our results suggest that the deprotonation of ABA by H16 ensures the stability of ABA inside the binding site and promotes the geometry between UPG and ABA that is more conducive to catalysis,, thus supporting the essential role of H16.

**Figure 14:**
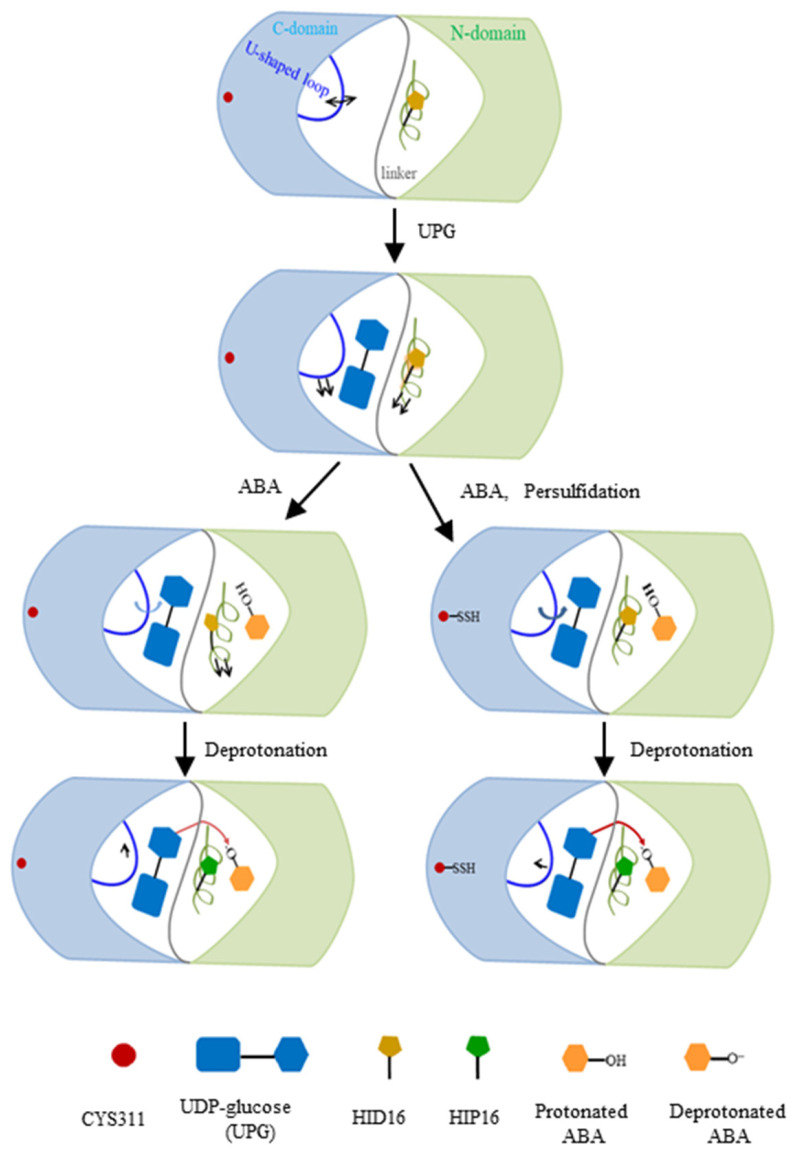
A scheme of ABA glycosylation by UGT71C5, as well as the regulatory role of persulfidation in this process. The N-terminal domain, colored in light green, and the C-terminal domain in light blue are connected by a gray linker in UGT71C5. The *α*-helix structure containing H16 in the N-terminal domain is colored in lime green and the U-shaped loop in the C-terminal domain is blue. The arrows, attached to the *α*-helix and the U-shaped loop, denote the movement direction of structural elements. Note that the direction change in the *α*-helix’s motion is in reference to the substrate-free state and the change in the U-shaped loop is in reference to the previous state. The shade of the blue arrow attached to the U-shaped loop is indicative of the motion amplitude, the deeper one to a larger amplitude of motion, and the shallower one to a smaller amplitude. The red arrow represents the transfer of the glycosyl moiety, with the darker red color corresponding to a facilitated moiety. The U-shaped loop structure displays distinct directional movements in response to various environments. When ABA binds to the sugar acceptor site, the U-shaped loop in the persulfidated UGT71C5 shifts more toward the glycosyl moiety compared to the non-persulfidated counterpart form. This slight positional change helps stablize the catalytic base, H16, through hydrogen bond interaction between H16 and UPG, preparing for ABA deprotonation. Once ABA is deprotonated, the hydrogen bond interaction weakens and the U-shaped loop repositions. The adjustments bring UPG and deprotonated ABA closer to a ‘Michaelis’ complex in the persulfidated UGT71C5, facilitating glycosyl moiety transfer. Consequently, the persulfidation of UGT71C5 promotes the reversible inactivation of ABA.

Comparative and quantitative proteomic analyses, used to detect persulfidated proteins in *Arabidopsis* leaves treated with exogenous ABA, revealed that UGT71C5 exhibited a significant reduction in persulfidation levels. Specifically, UGT71C5 showed a 4.31-fold decrease after 3 h of ABA treatment, which decreased to 2.20-fold after 6 h compared to the control condition [41]. These findings suggest that the persulfidation of UGT71C5 varies dynamically with the duration of ABA treatment, potentially implicating it in ABA signaling transduction pathways. This variability implies that UGT71C5’s persulfidation status may regulate its enzymatic activity, and thus influence the level of free ABA in plants. We thereby modeled two ternary complexes in which UGT71C5 was in a persulfidated state (persulfidated UGT71C5 complexed with UPG, and protonated ABA together with deprotonated ABA). The deprotonation of ABA by catalytic histidine is important for glycosylation. As our results show, in the case of protonated ABA binding to UGT71C5, persulfidation positions catalytic histidine (H16) optimally, which facilitates the deprotonation of ABA. Combining the data of RMSD calculations for the glycosyl moiety (Figure S5A) and hydrogen bonding interaction maps between UPG and UGT71C5 (Figure 6), we obtained information explaining the mentioned effect exerted by persulfidation. The RMSDs show that the positional fluctuation in the glycosyl moiety is larger in persulfidated systems relative to their non-persulfidated counterparts. This is probably because the movement of UPG establishes a hydrogen bond connection with H16, supporting H16 in the appropriate position, which prepares for deprotonation. After the deprotonation of ABA by H16, ABA is stable at the acceptor binding site, and the connection between UPG and H16 is also considerably weakened. Then, in the case of deprotonated ABA binding to UGT71C5, persulfidation assists in the reorientation of UPG and ABA closer to the ‘Michaelis’ configuration (Figure 10C), facilitating the transfer of the glycosyl moiety. Thus, persulfidation plays a part in the deprotonation of ABA and the transfer of the glycosyl moiety, and the model representation of the regulatory role played by persulfidation in UGT71C5 is shown in Figure 14 with a detailed explanation.

While the in silico simulations provide valuable insights into UGT71C5’s enzyme function through persulfidation, there are limitations to this approach. The simulations, although detailed, are based on theoretical models and assumptions that may not fully capture the complexities of in vivo conditions. Real-time protein dynamics, biomolecular interactions, and cellular environments are difficult to model accurately. The in vitro assays and recombinant protein expression experiments conducted, while supportive, are not exhaustive enough to confirm the findings definitively. Further experimental validation in physiological contexts and additional in vivo studies are necessary to corroborate the results and strengthen the conclusions of this research.

## 4. Materials and Methods

### 4.1. Recombinant Protein Expression and Site-Directed Mutagenesis

For in vitro protein expression, the complete coding region of UGT71C5 was amplified and inserted in frame into the plasmid pET28a-HIS. The recombinant HIS-tagged protein was purified from an *Escherichia coli* Rosetta (DE3) cell, with 16 °C and 18 h as the protein expression conditions. Site-directed mutagenesis was carried out using the Mut Express II Fast Mutagenesis Kit V2, following the manufacturer’s manual. All designed primers in this study are listed in Supplementary Table S1.

### 4.2. In Vitro Persulfidation Assay

Persulfidated proteins were detected using an MBST method [43]. In the in vitro persulfidation assays, the purified recombinant proteins were incubated with 250 μM of NaHS for 1 h to increase the persulfidated proteins or treated with 5 mM of TCEP for 1 h to reduce all of the disulfide bonds. The untreated proteins were used as a control. The free thiols of cysteines in proteins were initially blocked with the electrophilic alkylating agent, S-methyl methanethiosulfonate (MMTS), and the persulfidated cysteines were then labeled with biotin in the presence of N-[6-(biotinamido)hexyl]-39-(2′-pyridyldithio)-propionamide (Biotin-HPDP). The persulfidated proteins were detected by immunoblotting using an anti-biotin antibody. The total proteins were detected by immunoblotting using an anti-HIS antibody. The raw images are shown in Supplementary Figure S15.

### 4.3. System Setup

Molecular dynamics simulations were carried out for six systems: substrate-free UGT71C5, UPG-bound UGT71C5 complex, and UGT71C5 and persulfidated UGT71C5 complexed with UPG/ABA in deprotonated and protonated forms. The structures predicted by AlphaFold [51,52] and trRosetta [53] were integrated as the starting structure of UGT71C5. The integration replaced the region consisting of residues 1 to 123 from AlphaFoldby the counterpart from trRosetta2, which aids to release spatial clashes when docking ABA into UGT71C5. The binding of UPG within the donor substrate binding site was performed by referencing the structures downloaded from the Protein Data Bank (PDB codes: 2acw [49], 2vce [50], and 2c1z [28]). UGT71C5 was superimposed onto flavonoid 3-O glucosyltransferase (PDB code: 2c1z) to determine ABA binding at the acceptor substrate site. The initial structures of UGT71C5 complexed with both UGP and ABA were obtained by employing Schrödinger Maestro [54], considering both the deprotonation and protonation of catalytic histidine (H16). The persulfidated structures of UGT71C5 were obtained by modifying the targeted residue (C311) in the corresponding wild-type structures.

### 4.4. Conventional Molecular Dynamics (CMD) Simulation

For all systems, AMBER force fields ff14SB [55] was applied to the proteins, and the force-filed parameters of UPG and protonated and deprotonated ABA were generated based on the general AMBER force field (gaff) [56] by antechamber [57]. The restrained electrostatic potential (RESP) [58] approach was used to assign partial charges for these substrates based on the electrostatic potential calculated at the B3LYP/6-311G** level of theory. The user-defined force-field parameter of persulfidated cysteine in our previous study [59] was employed for this case. Each system was solvated in a cubic box filled with TIP3P waters [60] with a 10 Å buffer zone and neutralized by adding a total of 20 Na^+^ ions under a physiological pH.

Before starting the simulation, the solvated system was energy-minimized at four stages: the first three with all heavy atoms, the backbone atoms, and C*α* atoms restrained in turn imposing force constants of 5.0, 2.0, 1.0, and 0 kcal mol^−1^ Å^2^, respectively, and then without any restraint. Following these steps, the systems were then heated to 300 K in 250 ps, with non-hydrogen atoms being restrained by a force constant of 10 kcal mol^−1^ Å^2^. Next, each system underwent 4.5 ns of equilibration in two stages on the canonical (NVT) ensemble, applying restraints to non-hydrogen atoms with force constants of 1.0 and 0.5 kcal mol^−1^ Å^2^, and three stages on the isothermal–isobaric (NPT) ensemble, applying restraints to backbone and Cα atoms with force constants of 0.5, 0.1, and 0.1 kcal mol^−1^ Å^2^. The simulation was then continued with a 2 fs timestep on the NPT ensemble without constraints. The combination of the Langevin thermostat and Berendsen barostat was adopted to generate the NVT and NPT ensembles. Each system performed three replicated simulations through setting random seeds in AMBER18 [61]. All three parallel simulation times were 900 ns for UPT71C5 complexed with UPG, 700 ns for persulfidated UGT71C5 complexed UPG and protonated ABA, and 600 ns for the other UGT71C5 structures. During the simulations, the SHAKE algorithm [62] was used to constrain the bonds involving hydrogen. The particle-mesh Ewald sum method [63] was used for computing the Coulomb interactions under the periodic boundary condition, and a 10 Å distance cutoff value was used for non-bonded interactions.

### 4.5. Steered Molecular Dynamics (SMD) Simulation

SMD simulation [64,65,66] is a technique to manipulate the system from an initial configuration to a target configuration by applying an external force. In the present work, SMD simulations were performed to pull deprotonated ABA out of the sugar–acceptor binding site of UGT71C5 and persulfidated UGT71C5. The three equilibrated structures obtained from CMD simulations for each of the two UGT71C5 complexes served as the initial coordinates for the SMD simulations. And, three independent 2 ns simulations were performed for each equilibrated structure, resulting in a total of nine simulations for each complex. During the SMD simulation stage, a pulling force with a spring constant of 10 kcal mol^−1^ Å^2^ was applied to pull the product out of the active site, where the force along the line connecting the center of mass (COM) of the ABA and residues within 5 Å^2^ of deprotonated ABA.

### 4.6. Analysis of MD Trajectories

The CPPTRAJ tool [67] was used for general analyses, such as root mean square deviation (RMSD) calculations, root mean square fluctuation (RMSF) calculations, and correlation analyses. Scikit-learn version 1.0.2 and factor_analyzer version 0.4.1 with Python 3.10 were used for rotated principal component analysis (PCA). The RMSD calculation conducted separately for each replicate simulation was performed on C*α* atoms, with the first frame as a reference structure using whole production trajectories. The RMSF calculations on each parallel simulation were also performed on C*α* atoms, with the average structure of equilibrium trajectories as a reference structure. The rotated PCA followed in two steps: apply the traditional PCA to the trajectory dataset first, and then perform rotations on the obtained component values. The PCA [68] was carried out using C*α* atom coordinates obtained by concatenating equilibrated trajectories from all systems and conducting RMSD fitting. The isotropic reorientational eigenmode dynamics (iRED) method was used to calculate S^2^ order parameters for C*α*–C*β* vectors, excluding glycine. iRED [69] relies on the PCA of the isotropically averaged covariance matrix. The RMSD-based clustering of combined trajectories in each system was performed using the DBSCAN algorithm [70]. The proportion of each cluster was calculated, and the similar conformations in the trajectories were divided into the same cluster after clustering.

## 5. Conclusions

In the present study, the MBST method was used to determine the persulfidation site of UGT71C5, and CMD and SMD simulations were applied to analyze the conformational and dynamical changes in UGT71C5 related to functional response, as well as the role played by persulfidation. The persulfidation assays indicate that C311 is a modification site. Based on the CMD trajectories, the analyses indicated that the linker has the capability of inducing conformational rearrangements in the UPG binding site, which mainly depends on its composition. R258 situated at the linker undergoes side-chain reorientations in response to UPG binding, likely due to the hydroxyl groups in UPG, which causes further conformatiional changes in its surroundings. In addition, the U-shaped loop functions as a regulatory structural element associated with the binding of UPG and ABA. Additionally, the plasticity of the U-shaped loop allows persulfidation to help stabilize the critical catalytic residue H16, facilitating the deprotonation of ABA, and then aids in repositioning UPG and IAA closer to the ‘Michaelis’ configuration, thereby favoring the formation of ABA-GE.From the SMD results, it was suggested that the persulfidation of UGT71C5 can lead to a more efficient release of the sugar–acceptor inferred by the reduced work required and alternative paths in persulfidated UGT71C5, which is also presumably true for the conjugated sugar–acceptor. Together, the CMD and SMD results highlight the role of persulfidation, that is, persulfidation facilitates the reversible inactivation of ABA. Overall, this work identified the specific persulfidation site of UGT71C5, as well as the important structural elements participating in the substrate recognition of UGT71C5, and provided some insights into the persulfidation of UGT71C5, which might be a mechanism of H_2_S modulating ABA level. Thus, an understanding of the molecular basis presented here is helpful to guide the modulation of ABA level.

## Data Availability

Data are contained within the article and Supplementary Materials.

## Supplementary Materials

The following supporting information can be downloaded at: https://www.mdpi.com/article/10.3390/ijms25179679/s1.
