# Supplementary material for: Insights from Structure-Based Simulations into the Persulfidation of Uridine Diphosphate-Glycosyltransferase71c5 Facilitating the Reversible Inactivation of Abscisic Acid
Source: Int J Mol Sci. 2024 Sep 6;25(17):9679. doi: 10.3390/ijms25179679 (PMC11395816; doi:10.3390/ijms25179679)
Supplement: Supplementary file 1 [file ijms-25-09679-s001.zip › ijms-3159487-supplementary.pdf]

Article

Insights from Structure-Based Simulations into the Persulfidation of Uridine Diphosphate-Glycosyltransferase71c5 Facilitating the Reversible Inactivation of Abscisic Acid

Miaomiao Li [1,2], Lihui Xiao [1], Ke Sun [1], Taotao Qiu [1], Sisong Lai [1], Guojing Chen [1], Lingxi Geng [1], Siqi Huang [2,*] and Yanjie Xie [1,2,*]

1 College of Life Sciences, Nanjing Agricultural University, Nanjing 210095, China; 2021216040@stu.njau.edu.cn (M.L.); 2023116078@stu.njau.edu.cn (L.X.); 9201010221@stu.njau.edu.cn (L.G.)

2 Institute of Bast Fiber Crops, Chinese Academy of Agricultural Sciences (IBFC, CAAS), Changsha 410221, China

* Correspondence: huangsiqi@caas.cn (S.H.); yjxie@njau.edu.cn (Y.X.)

Table S1 PCR primers used in this study.

Primer	Sequence
UGT71C5(F)	gtgccgcgcggcagccatatgATGAAGACAGCAGAGCTCATATTCG
UGT71C5(R)	gtggtggtggtggtgctcgagTCAAAAGTGATCCCCAAGAATATCT
UGT71C5-C311S(F)	TATCGGGtccAGGTTCATCTGGGCGATCCGTA
UGT71C5-C311S(R)	TGAACCTggaCCCGATAAGCTCGAGCGCGTGA

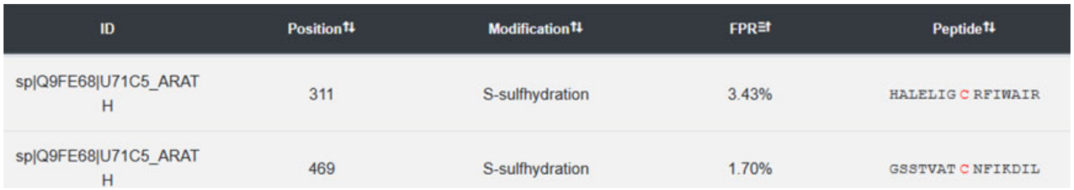

ID	Position	Modification	FPR	Peptide
sp|Q9FE68|U71C5_ARATH	311	S-sulfhydration	3.43%	HALELIG C RFIWAIR
sp|Q9FE68|U71C5_ARATH	469	S-sulfhydration	1.70%	GSSTVAT C NFIKDIL

Figure S1: Prediction about potential cysteine modification site(s) was done on the server http://pcysmod.omicsbio.info/ using UGT71C5 sequence.

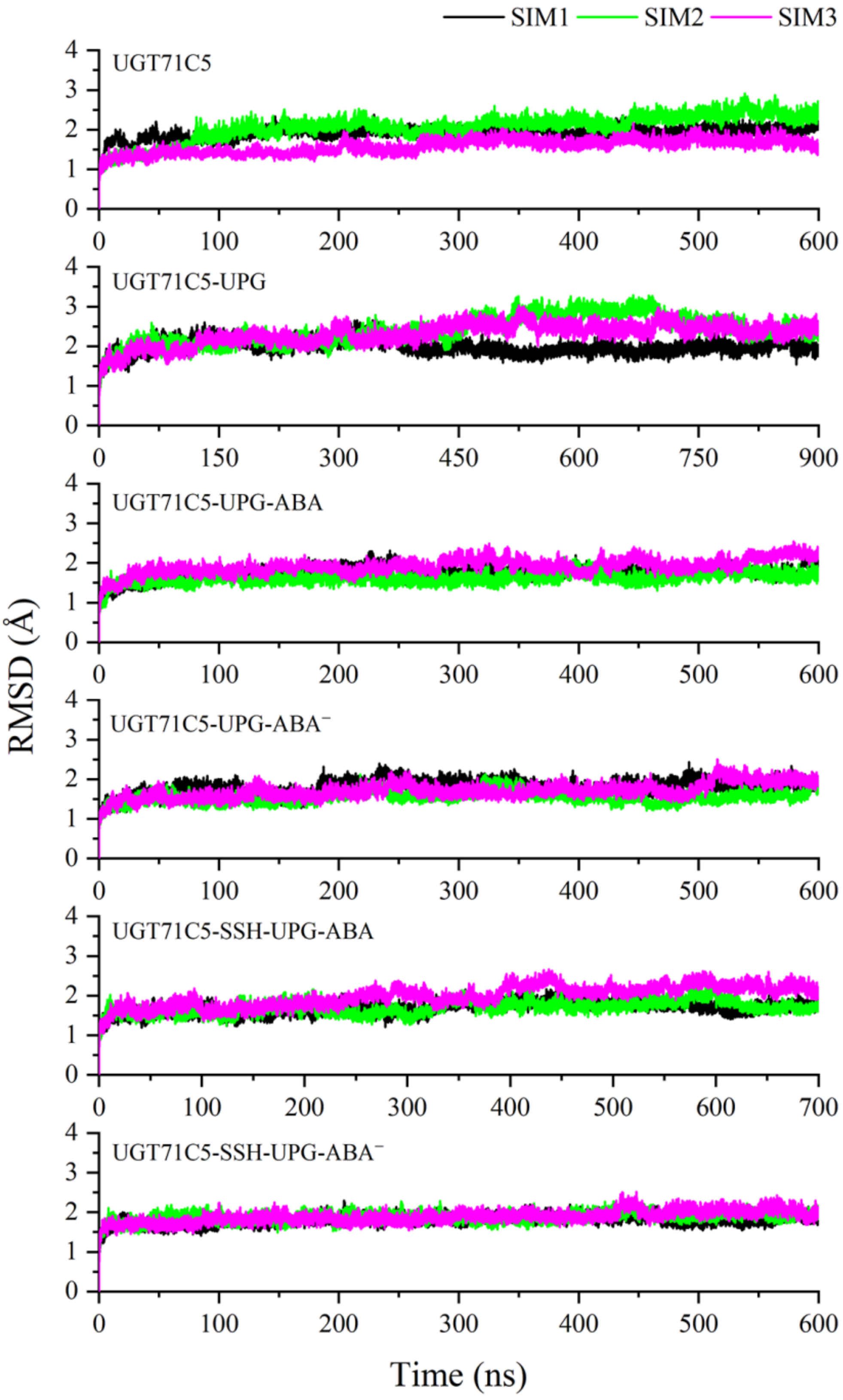

Figure S2: The Cα root-mean-square deviations (RMSDs) of UGT71C5s. RMSDs as a function of simulation time.

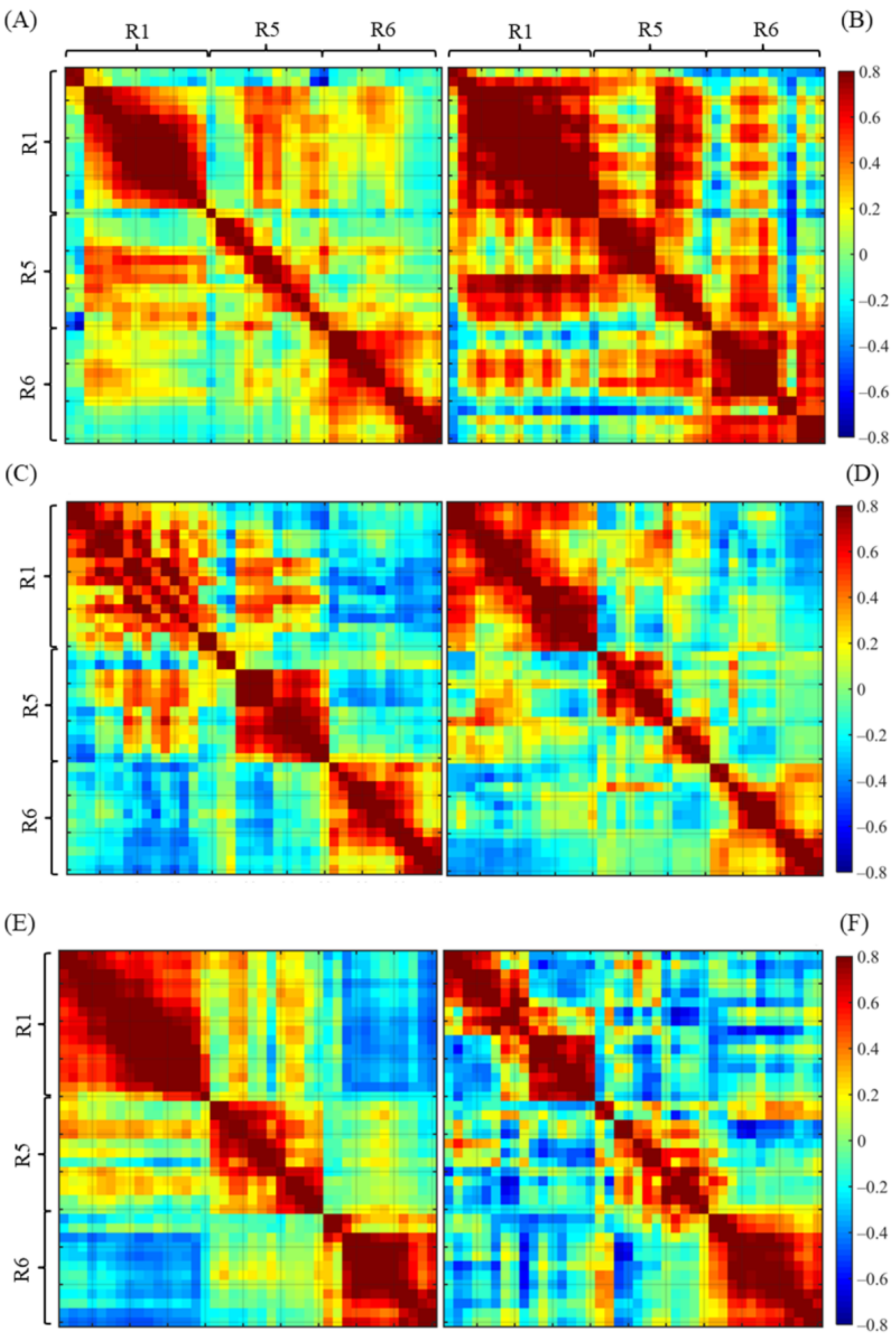

Figure S3: The covariance matrix maps of three regions (R1, R5, and R6) of UGT71C5 in each system. (A) UGT71C5; (B) UGT71C5-UPG; (C) UGT71C5-UPG-ABA; (D) UGT71C5-UPG-ABA^{-}; (E) UGT71C5-SSH-UPG-ABA; (F) UGT71C5-SSH-UPG-ABA^{-}.

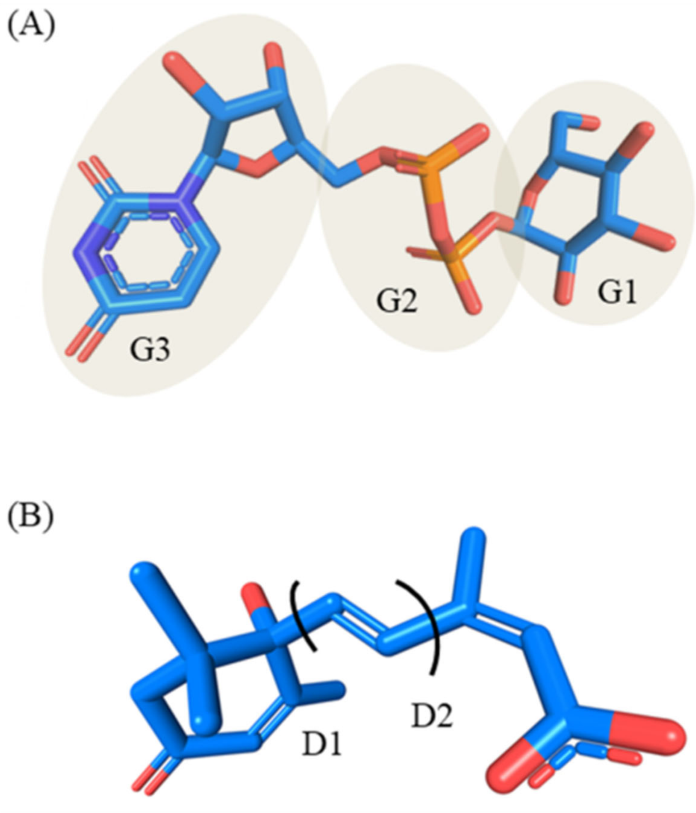

Figure S4: Molecular representation of two substrates (UPG and ABA) of UGT71C5. (A) The UPG is broken into three moieties: glycosyl (G1), phosphate (G2), and uridine (G3), respectively. (B) Two rotatable bonds (denotations D1 and D2) with black arc labelled are used for defining the movement of ABA.

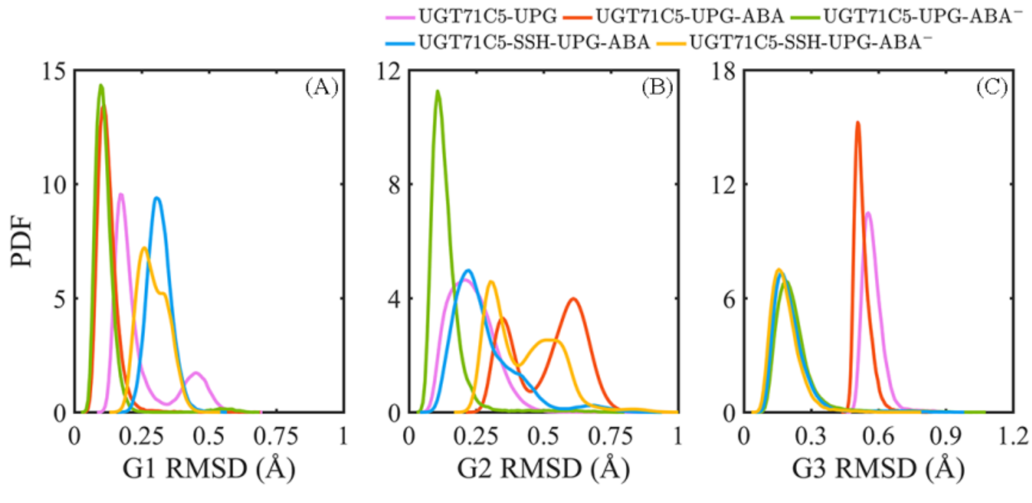

Figure S5: The probability distribution function (PDF) of RMSDs of moiety atoms: (A) glycosyl moiety, (B) phosphate moiety; (C) uridine moiety according to their average coordinates in different complex systems during the last 3 × 150 ns simulations.

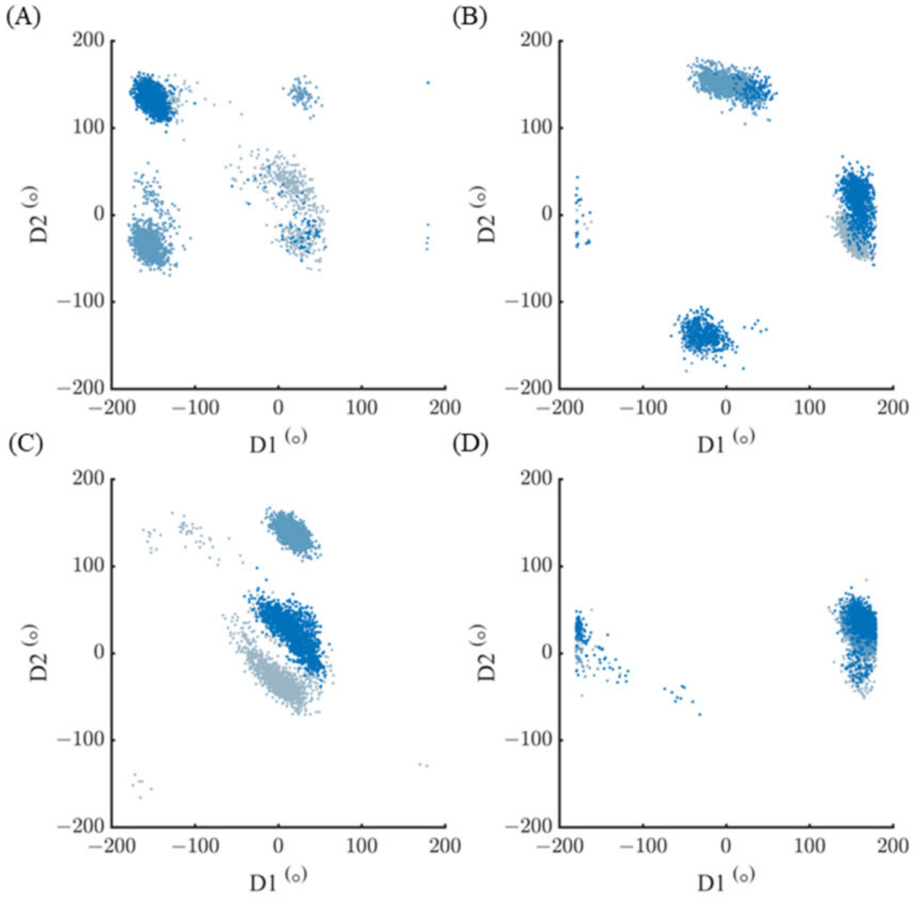

Figure S6: Joint distribution of the dihedral angles of ABA in ternary UGT71C5 complexes. Data were extracted every 100 ps of 3 × 150 ns equilibrated production simulations.

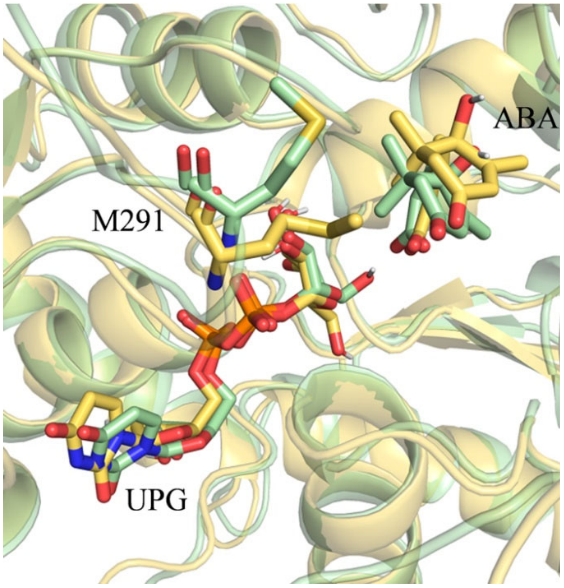

Figure S7: Comparison of substrate binding sites within UGT71C5 between UGT71C5-UPG-ABA⁻ and UGT71C5-SSH-UPG-ABA⁻ systems. Non-persulfidated UGT71C5

is shown in pale green and persulfidated one is shown in pale yellow. UPG and ABA together with residue M291 in the U-shaped loop are in stick representations.

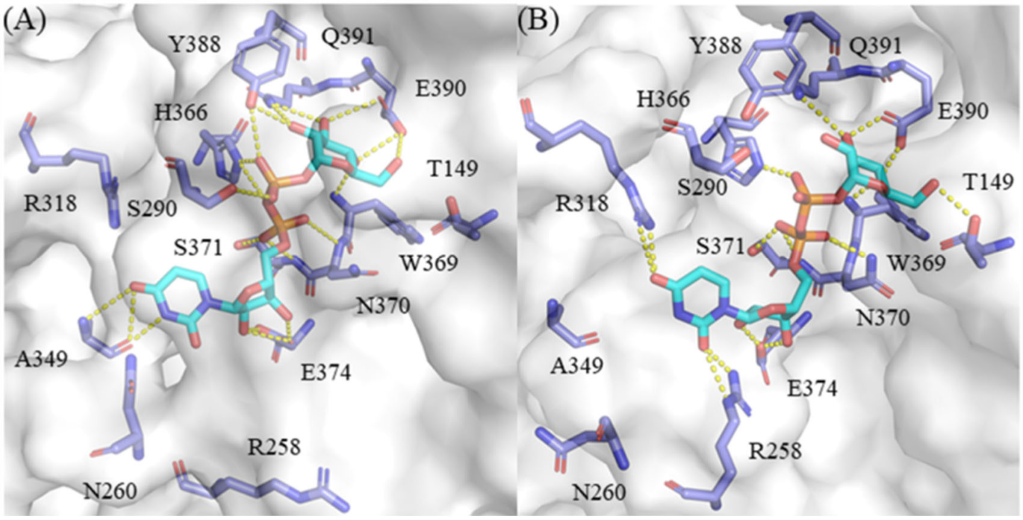

Figure S8: Hydrogen bond connections between UPG and UGT71C5 in (A) starting structure and (B) the representative structure from UGT71C5-UPG-ABA^{-} system. Yellow dash line represents the hydrogen bond connection. UGT71C5 residues and UPG are in stick representation.

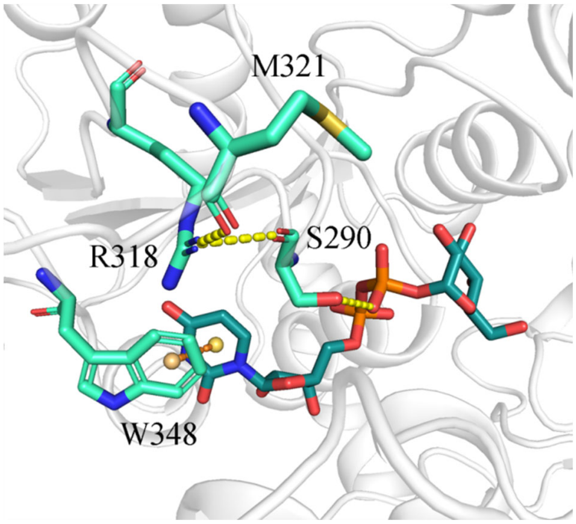

Figure S9: Intra- and inter-molecular interactions within UPG binding site. The side-chain nitrogen atom of R318 forms hydrogen bonds with main-chain carbonyl oxygen atom of M321 and S290, in which S290 also forms hydrogen bond with UPG. Additionally, a π-π stacking interaction formed between W348 and UPG.

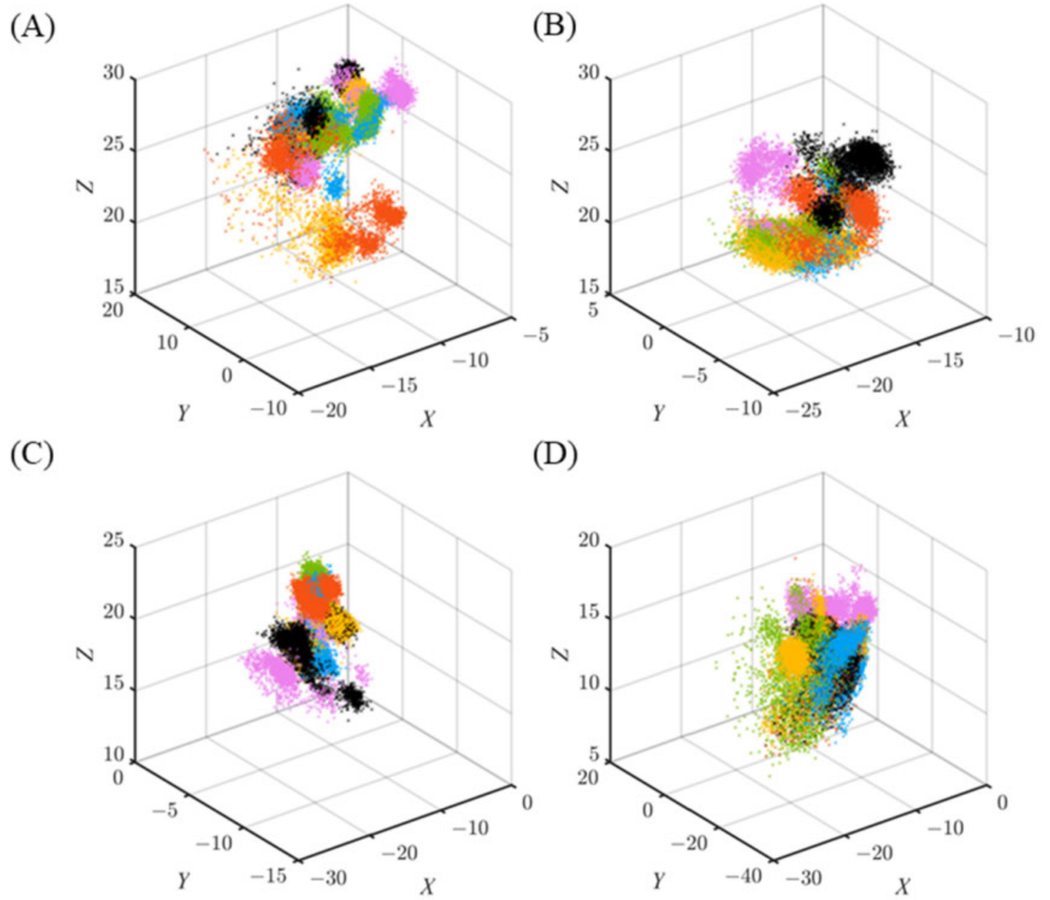

Figure S10: Distribution of the side-chain centers of four residues in 3D space of each system: R258 (A); W348 (B); R318 (C); and M291 (D). The sampled positions are shown in dots with matching color. Black: UGT71C5 system; magenta: UGT71C5-UPG system; darkorange: UGT71C5-UPG-ABA system; forestgreen: UGT71C5-UPG-ABA^{-} system; deepskyblue: UGT71C5-SSH-UPG-ABA system; goldenord: UGT71C5-SSH-UPG-ABA^{-} system.

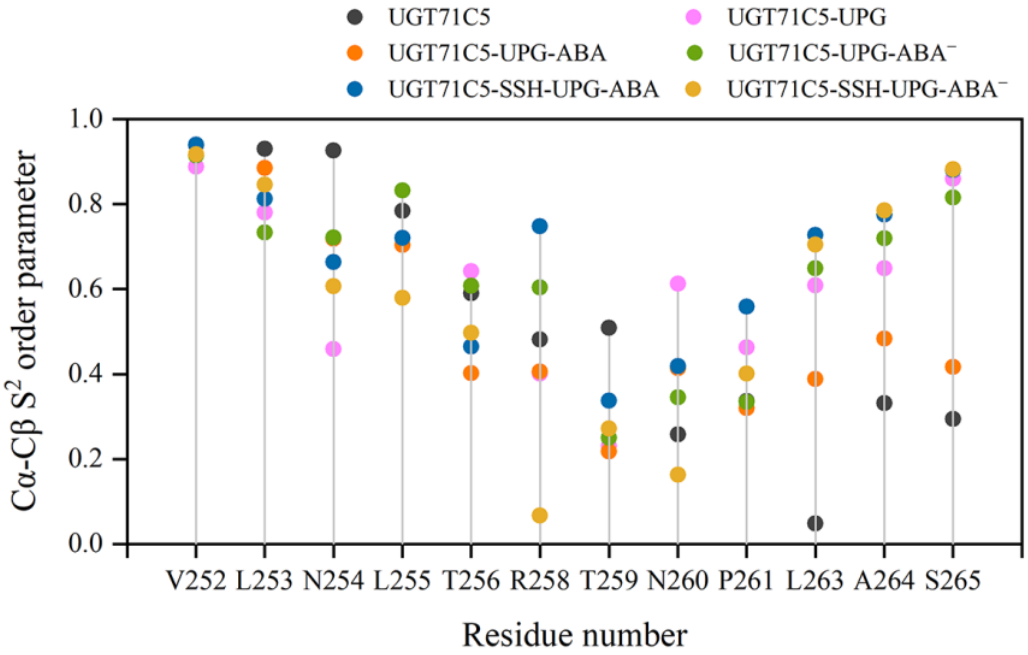

Figure S11: The S^2 order parameter for Cα-Cβ bond vector of residues in linker loop.

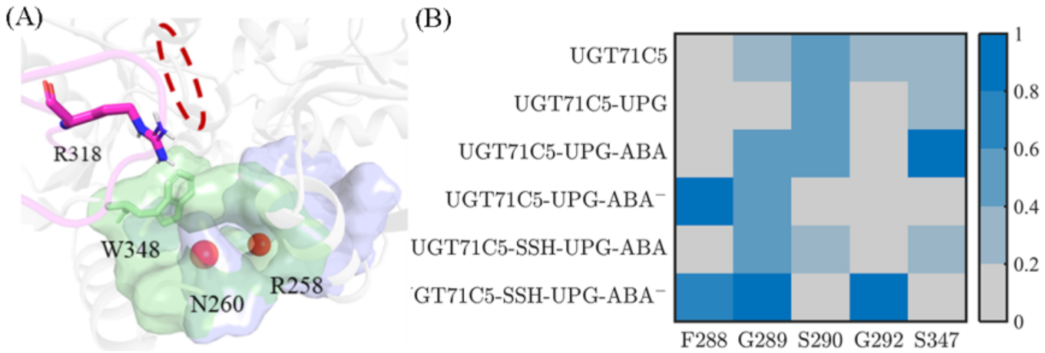

Figure S12: (A) The spatial location information of several regions and important residues in UGT71C5. The U-shaped loop is circled by a deepred oval and the disordered loop is colored in magenta. R318 and W348 are displayed by stick in magenta and palegreen, respectively. R258 and N260 are shown in red sphere. Molecular surface in lavender blue represents the surrounding within 4 Å of R258, and the palegreen one corresponds to N260. (B) Hydrogen-bonding interaction network involving in R318. The color indicates the proportion of a hydrogen bond during the simulation.

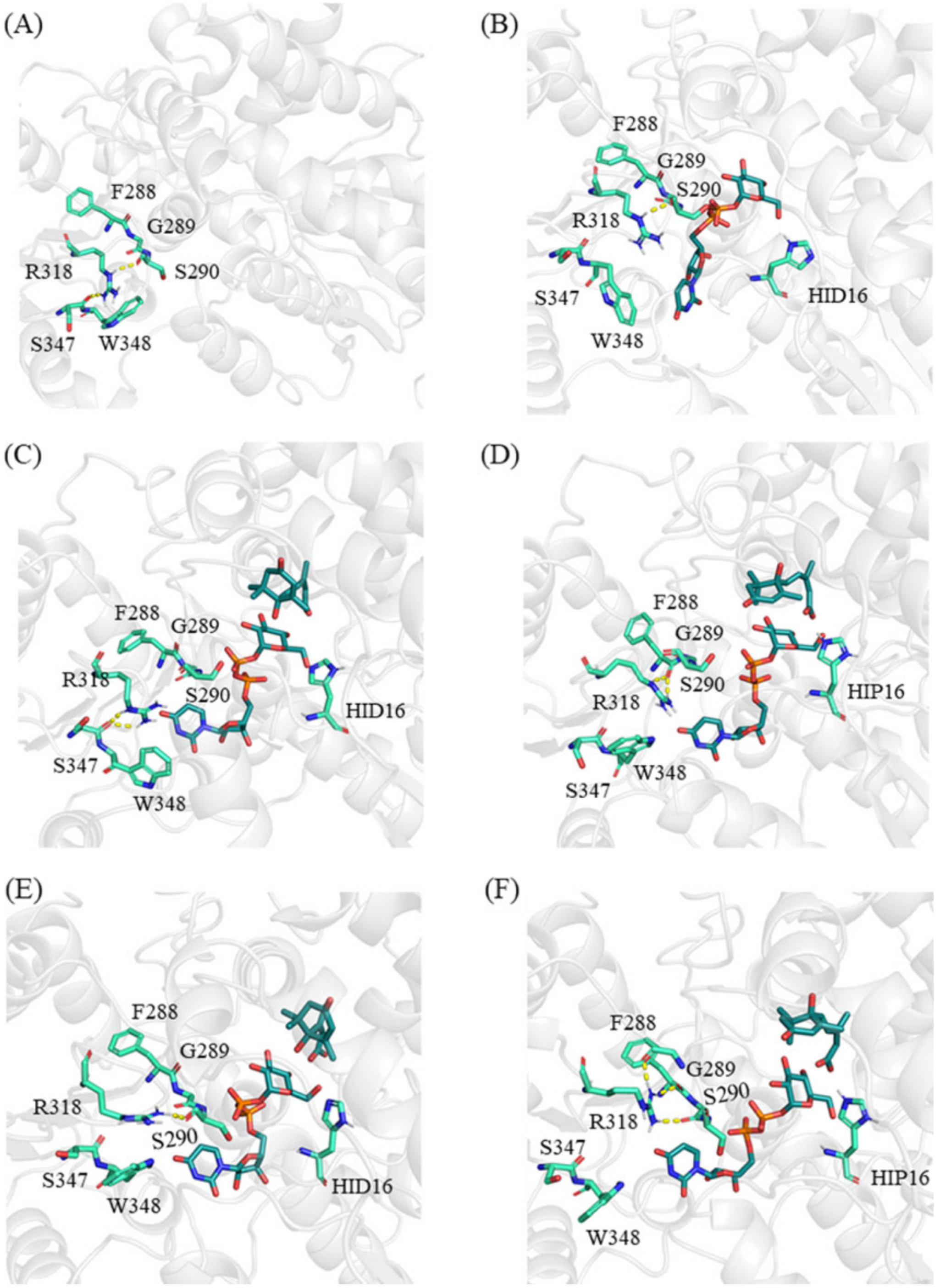

Figure S13: Local hydrogen-bonding interaction network involving in R318 and several residues around in representative structure of UGT71C5s. The residues are shown in greencyan stick and two substrates (UPG and ABA) are shown in teal stick. (A) UGT71C5 system; (B) UGT71C5-UPG system; (C) UGT71C5-UPG-ABA system; (D) UGT71C5-UPG-ABA^{-} system; (E) UGT71C5-SSH-UPG-ABA system; (F) UGT71C5-SSH-UPG-ABA^{-} system.

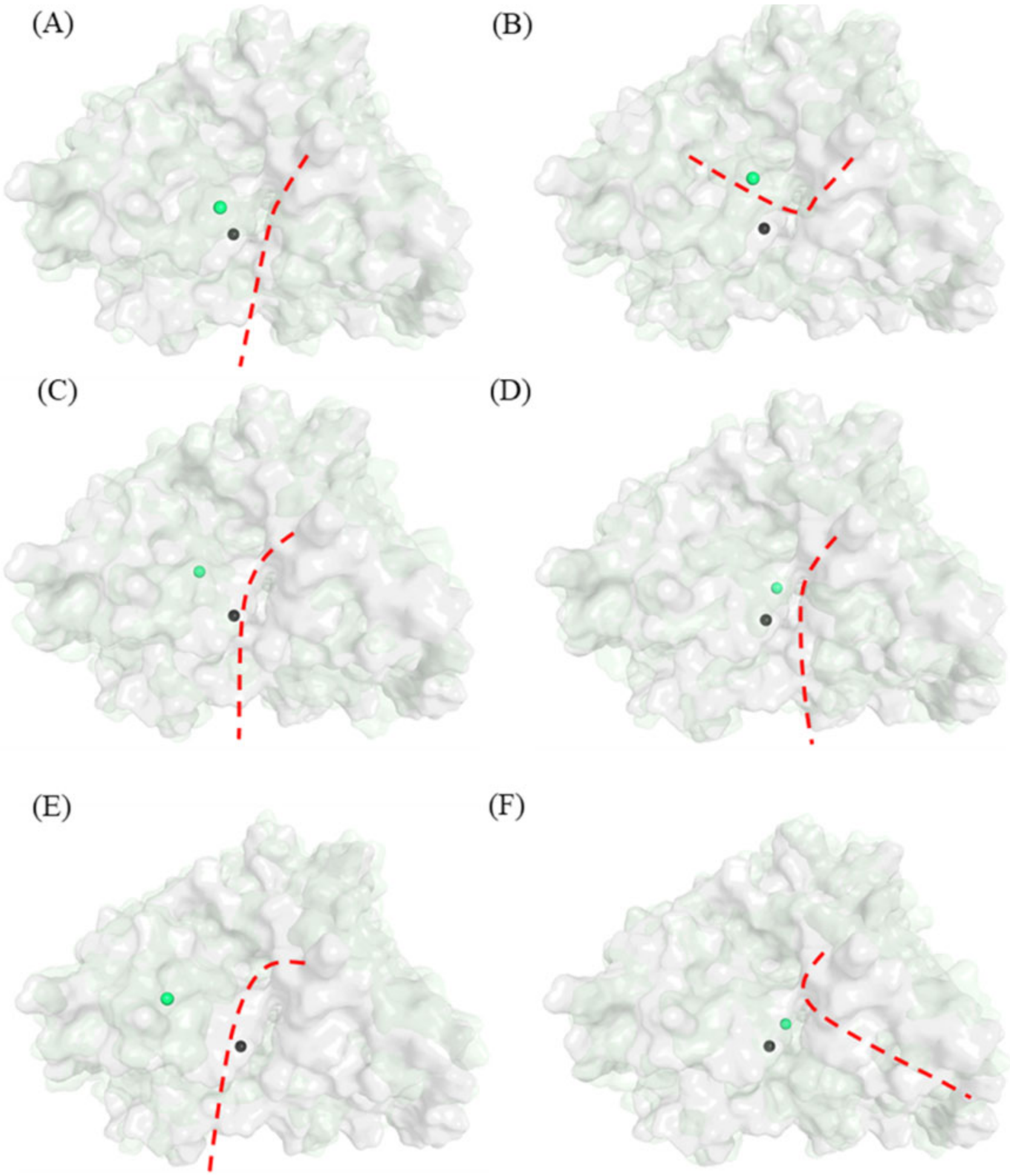

Figure S14: Schematic plot of the release pathways for ABA: (A-C) UGT71C5-UPG-ABA^{-} system and (D-F) UGT71C5-SSH-UPG-ABA^{-} system. White molecular surface representation for the starting structure of UGT71C5 applied in CMD and limegreen molecular surface representation for the one applied in SMD. The position of the disordered loop in UGT71C5 is roughly characterized by its glycine. The starting position of glycine is represented by a black sphere and the position after dynamic change by limegreen sphere.

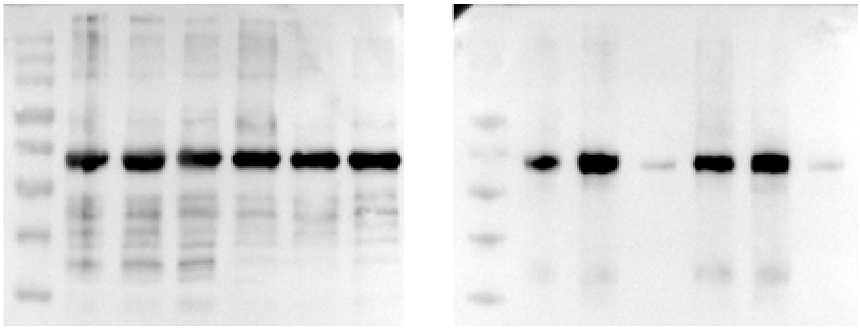

Figure S15: The raw images of immunoblotting related to persulfiadation assays.
